# Cyber Defense Effectiveness Evaluation for ICS Under Uncertainty: A Dynamic Bayesian Network Approach with Information Entropy

**DOI:** 10.3390/e28060635

**Published:** 2026-06-04

**Authors:** Rongbao Kang, Zhiyong Zhang, Xiao Zhang, Jianfeng Chen, Ruoyu Xu, Yongdong Zhang, Zhihong Rao

**Affiliations:** 1 School of Cyberspace Security, University of Science and Technology of China, Hefei 230026, China; krbhl@mail.ustc.edu.cn (R.K.); zhyd73@ustc.edu.cn (Y.Z.); 2 No. 30 Research Institute of China Electronics Technology Group Corporation, Chengdu 610041, China; zhiyong_zhang1013@163.com (Z.Z.); iamzhangxiao@126.com (X.Z.); atrix@163.com (J.C.); 3 School of Cyberspace Security, Northwestern Polytechnical University, Xi’an 710072, China; xuruoyu@mail.nwpu.edu.cn

**Keywords:** industrial control systems, dynamic Bayesian network, information entropy, cyber defense effectiveness, epistemic uncertainty, aleatoric uncertainty, risk-aware decision-making

## Abstract

Proactive defense planning in Industrial Control Systems (ICS) is critically constrained by two intertwined types of uncertainty: epistemic uncertainty, arising from the defender’s limited observability of the system state and incomplete knowledge of attacker strategies, and aleatoric uncertainty, stemming from the stochastic nature of state transitions and the propagation of disturbances through inter-device dependencies. These factors significantly complicate the quantitative assessment of defense strategies before deployment. To address this challenge, this study proposes a Dynamic Bayesian Network (DBN)-based framework that explicitly models four sources of uncertainty. Within this framework, the expectation of the effectiveness differential is coupled with its information entropy to jointly quantify expected performance and prediction uncertainty. A casestudy on a typical substation automation system, complemented by systematic ablation experiments, demonstrates that the framework can effectively distinguish the relative effectiveness of defense strategies. The framework maintains robust assessment results under up to 15% noise in Conditional Probability Tables (CPTs). The ablation experiments further quantify the individual contributions of observability, dependency propagation, and attacker strategy to prediction uncertainty, and reveal a non-trivial coupling between epistemic and aleatoric uncertainty. This research provides theoretical and methodological support for resilience-oriented cyber defense planning in ICS.

## 1. Introduction

ICS serve as the core infrastructure for critical sectors such as energy, water, and transportation, making their security directly linked to public safety. However, with the widespread adoption of industrial internet of things technologies, cyber threats against ICS have evolved from simple attacks to multi-stage, targeted penetration and integrated cyber–physical attacks, as exemplified by Stuxnet [1] and the 2015 Ukrainian power grid attack [2]. The frequency and severity of such attacks continue to escalate [3,4]. Unlike conventional IT systems, post-attack recovery for ICS is often irreversible or extremely costly. Therefore, proactive defense planning—evaluating and optimizing strategies in the pre-engagement phase—has become more critical than post-incident analysis. Defenders thus require quantitative tools that can both predict the effectiveness of alternative strategies and assess the confidence of such predictions under uncertainty. However, this task is fundamentally constrained by two intertwined challenges: (i) the perspective bias in the defender’s observations and knowledge of attacker strategies (epistemic uncertainty), and (ii) the inherent randomness in system state evolution driven by attack–defense interactions and device interdependencies (aleatoric uncertainty). A recent systematic review on risk and reliability evaluation confirms that traditional methods are predominantly static and highlights the urgent requirement for dynamic, model-based techniques to address the rising complexity and heterogeneity of future industrial automation systems [5].

Extensive research has addressed these challenges in three main directions: probabilistic and dynamic risk assessment, attack–defense modeling, and uncertainty quantification.

**Probabilistic and dynamic risk assessment in ICS.** Traditional static approaches, such as CVSS-based scoring, are limited to snapshot assessments and cannot capture temporal state evolution. Bayesian network (BN)-based methods have therefore been widely adopted. Recent static BN advances include frameworks combining AutomationML with Bayesian Networks for safety–security assessments [6], and probabilistic models linking machine-level configurations to security incidents for decision support [7]. On the dynamic front, DBNs and related temporal models capture state evolution under adversarial impacts. Representative works include proactive dynamic risk assessment integrating internal data and threat intelligence for SCADA systems [8], and Bayesian Attack Graphs (BAGs) that combine network topology with CVSS data for dynamic risk evaluation [9], sharing temporal inference capabilities with DBNs. A fundamental limitation persists across these works: they assume the evaluator has complete system knowledge—a global perspective rarely available in adversarial environments.

**Attack–defense modeling and game-theoretic approaches.** To capture strategic attacker–defender interactions, game-theoretic models, Markov decision processes, and optimization frameworks have been developed. These include attacker–defender game models with constrained strategies that employ information theory to evaluate the diversity and unpredictability of strategy selections [10]. In that work, entropy is used to capture a single dimension of strategic uncertainty, which is distinct from the comprehensive multi-source uncertainty aggregation proposed here. Other representative works in this stream include game-theoretic frameworks for cross-layer impact assessment in industrial cyber–physical systems [11], optimization approaches generating worst-case attack strategies [12], and Bayesian ATT&CK Networks (BANs) for data-driven APT attack prediction [13]. Recent work has also advanced attack detection within Bayesian Attack Graphs (BAGs) under realistic uncertainties: Kazeminajafabadi et al. [14] formulate the BAG as a hidden Markov model and derive an MMSE detector that explicitly accounts for imperfect monitoring and uncertain reimaging outcomes. While this work relaxes the full-observability assumption in attack detection, it remains focused on state estimation rather than pre-engagement strategy evaluation, and does not address the broader challenge of comparing alternative defense strategies under the combination of dual-uncertainty modeling and ICS-specific dependency propagation. Across these works, the defender’s information state is typically either assumed to be complete or limited to the detection of attack events through intrusion detection systems. The defender’s incomplete knowledge of the true system state—i.e., the epistemic uncertainty arising from the discrepancy between what is observed and what is actually true—is not yet widely incorporated as a structural component of the evaluation framework, which limits the applicability of these methods in pre-engagement scenarios where strategies must be assessed under partial knowledge of the current system configuration and device health. Pre-engagement defense evaluation—where defenders must decide under partial observations and quantify strategy impacts through dependency propagation—remains underexplored.

**Uncertainty quantification in cyber defense evaluation.** Methods based on information entropy, fuzzy theory, and probabilistic decision processes have been applied to cybersecurity. Entropy-based approaches have been combined with triangular fuzzy numbers for cross-domain ICS risk assessment [15]. Bayesian Networks have been integrated with expert elicitation to distinguish attacks from accidental failures under incomplete information [16]. Differential game frameworks have been proposed for APT defense on edge nodes [17]. POMDPs have also been employed for decision-making under partial observability. However, these approaches remain isolated: existing frameworks either model structural dependencies under full observability (e.g., DBNs) or handle partial observability without system-wide dependency propagation (e.g., POMDPs). No existing framework jointly models both uncertainty sources within a single model: DBN-based methods capture dynamic evolution yet assume full observability [18], while POMDP-based methods address partial observability yet lack dependency propagation [19]. Furthermore, existing studies rely on expected values or variance to characterize effectiveness outcomes, yet these metrics are tied to the numerical encoding of system states and do not provide a consistent measure of prediction uncertainty across different system configurations. Although entropy naturally aggregates multi-source uncertainty into a single scalar, it has not been integrated with dynamic probabilistic models for pre-engagement defense planning.

The above analysis reveals three critical gaps for pre-engagement ICS defense planning, echoing challenges diagnosed by recent reviews: risk analysis methods lag behind the sophistication of modern cyber–physical systems and must be extended toward dynamic, probabilistic techniques [5].

1. **Neglect of defender perspective limitation.** Most frameworks assume complete information, failing to model the discrepancy between observed and true system states—i.e., epistemic uncertainty. This yields overly optimistic evaluations disconnected from the defender’s actual environment.
2. **Lack of joint dual-uncertainty modeling.** Existing methods address epistemic or aleatoric uncertainty in isolation, without a framework that jointly models both within a single temporal probabilistic model. This fragmented treatment prevents defenders from simultaneously accounting for what they do not know about the current system state and what they cannot predict about its future evolution—a gap that directly motivates the need for a joint dual-uncertainty representation.
3. **Absence of an encoding-independent companion metric for prediction uncertainty.** Current approaches rely on expected values or variance to characterize effectiveness outcomes. Yet these metrics are tied to the numerical encoding of system states and do not provide a consistent, encoding-independent measure of prediction uncertainty. While information entropy offers such a measure, its integration with dynamic probabilistic models for pre-engagement defense evaluation remains an open gap.

The core problem is thus as follows: how to achieve the accurate, risk-aware evaluation of defense strategy effectiveness in pre-engagement ICS scenarios, under the dual constraints of perspective bias (epistemic uncertainty) and state evolution (aleatoric uncertainty), while providing an encoding-independent measure of total prediction uncertainty?

To address this, we propose a DBN-based evaluation framework grounded in a key insight: under uncertainty, defense effectiveness evaluation is no longer computing a deterministic difference between two known states. Rather, both current and future states become random variables—the former due to partial observability, the latter due to unknown attacker strategies and the stochastic nature of state evolution. The problem thus transforms into characterizing the joint distribution of successive states, conditioned on the defender’s observations and chosen strategy. This formulation maps directly onto the inference structure of a DBN, providing a principled and computationally tractable basis for the evaluation framework. The main contributions are as follows:

1. **Joint DBN-based framework for dual-uncertainty representation.** We develop a DBN framework that simultaneously (i) embeds the defender’s partial observability and incomplete knowledge of attacker strategies (epistemic uncertainty), and (ii) models dynamic state evolution and inter-device dependency propagation (aleatoric uncertainty). By conditioning strategy evaluation on the defender’s actual observations rather than on an idealized global viewpoint, this framework provides a probabilistic representation that integrates both uncertainty types within a single temporal model.
2. **Dual-metric methodology with entropy as a companion metric.** Leveraging the DBN’s capacity to infer the full joint distribution of successive states, we couple the expected effectiveness differential with the information entropy of its predictive distribution. Operating in the probability domain rather than the numerical domain of variance, this entropy metric provides an encoding-independent measure of prediction uncertainty that captures the shape of the effectiveness distribution. Together, the two metrics jointly quantify anticipated performance and its associated unpredictability, offering a principled basis for risk-aware pre-engagement decisions.

These contributions collectively address all three identified gaps. The remainder of this paper is organized as follows. Section 2 presents the uncertainty modeling, the DBN formulation, the entropy-based effectiveness metrics, and the computational framework for deriving the dual-metric evaluation. Section 3 demonstrates the framework through a substation automation case study, including robustness analysis and the mechanistic interpretation of the experimental findings. Section 4 provides a systematic qualitative comparison with existing methods, discusses the choice of entropy as a companion metric, addresses parameter feasibility, and outlines limitations and future work. Section 5 concludes.

## 2. Methods

This section aims to construct a comprehensive probabilistic model of cyber attack–defense confrontation and to develop the associated effectiveness evaluation methodology. It captures the dynamic interactions among the attacker, defender, and the target network system, as well as the uncertainties inherent in the process. It is crucial to emphasize that the ultimate purpose of building this overall model is to provide a mathematical foundation for the core task of this work—effectiveness evaluation from the defender’s perspective prior to engagement. The following subsections will present the uncertainty modeling, the DBN formulation, and the computational framework for effectiveness evaluation including the expectation and information entropy metrics.

The network attack–defense process is an adversarial game between the attacker and defender centered on the target network. The uncertainty in the outcome of attack and defense actions primarily stems from two sources: the inherent observational uncertainty due to the limited perspective of both sides, and the intrinsic randomness in the evolution of the target network’s state. These two types of uncertainty are modeled separately below.

### 2.1. Uncertainty from Perspective Bias

In cyber attack–defense confrontations, both the attacker and defender operate in an environment characterized by information incompleteness, which leads to a fundamental perspective bias in their respective cognitions. Perspective bias is a core source of decision-making uncertainty. Consequently, we refer to the uncertainty arising from this bias as epistemic uncertainty and further decomposes it into two dimensions:

**Uncertainty in the observed state:** This refers to the probabilistic deviation between the state information perceived by either party from their own viewpoint and the true state of the system.

**Uncertainty in the adversary’s strategy:** This refers to the cognitive ambiguity one party faces regarding the potential strategies the opponent may adopt, due to the inability to directly observe the opponent’s decision-making process.

This subsection formalizes the perspective bias of both attack and defense parties. We emphasize that our model aims to objectively describe and characterize the existence and probabilistic nature of this bias, rather than to presuppose the superiority or inferiority of either party’s decisions.

#### 2.1.1. Uncertainty in Observed State

Let the multi-dimensional random vector $X=(X_{1},X_{2},\ldots ,X_{I})$ represent the true state of the target. Denote by $\Omega_{X}$ the sample space of X, i.e., the set of all possible states of the target. Note that each component $X_{i}\left(i=1,2,\ldots ,I\right)$ of X describes the state of a particular aspect of the target, which could be either a continuous or discrete random variable, depending on its physical meaning. While the framework can naturally extend to continuous variables through standard techniques such as probability density functions and integral-based inference, the formal exposition and the case study in this paper adopt a discrete state space to maintain clarity and to align with ICS risk assessment practice, where device states are typically classified into discrete severity levels.

Similarly, let the multi-dimensional random vectors $Y=(Y_{1},Y_{2},\ldots ,Y_{J})$ and $Z=(Z_{1},Z_{2},\ldots ,Z_{K})$ represent the target state perceived by the attacker and defender from their respective perspectives, related to X. Denote their sample spaces by $\Omega_{Y}$ and $\Omega_{Z}$, respectively.

Due to the adversarial nature of the game, the perceptions of the target state by the attacker and defender, Y and Z, are constrained and potentially interfered with, and thus are not identical to the true target state X. Moreover, the number of state parameters and even their physical meanings in Y and Z might differ from those in X. Therefore, both the attacker and defender can only infer the true network state X based on the correlation between their respective observed states (i.e., Y, Z) and X. Let the conditional probabilities P(X|Y) and P(X|Z) represent the inferred beliefs about the true target state X by the attacker and defender, respectively, given their observations Y and Z. According to Bayes’ theorem

$$P\left(X|Y\right)=\frac{P(Y|X)P(X)}{P(Y)}$$

$$P\left(X|Z\right)=\frac{P(Z|X)P(X)}{P(Z)}$$

Here, P(Y|X) and P(Z|X) are the likelihood probabilities of the attacker and defender observing Y and Z, respectively, given the true target state X.

**Remark on the dimensionality of observation vectors.** In practice, the strength of the correlation between Y, Z, and X reflects the reconnaissance and perception capabilities of the attacker and defender regarding the target network state. For instance, in the extreme case where Y and X are independent, the attacker gains no information about the target network state and the uncertainty in inferring X from Y is maximized. In typical ICS environments, the defender possesses higher access privileges and richer monitoring methods, so the correlation between Z and X is often stronger than that for the attacker. Beyond correlation strength, the observation vectors may also differ in dimensionality from the true state vector—i.e., J≠I and K≠I in the general formulation—reflecting the fact that the two parties monitor different, possibly overlapping, subsets of system attributes at different granularities. Accordingly, in the Bayesian network structure, each observation component $Z_{k}$ is modeled as depending on a known subset of the true state variables, i.e., $par(Z_{k})\subseteq X$. This structural flexibility allows the framework to accommodate arbitrary observation mappings without constraining the dimensionality of Z or Y to match that of X.

#### 2.1.2. Uncertainty in Adversary Strategy

Consider *M* possible attack strategies available to the attacker and *N* possible defense strategies available to the defender. Let the random vectors $A=(A_{1},A_{2},\ldots ,A_{M})$ and $D=(D_{1},D_{2},\ldots ,D_{N})$ characterize the strategy combinations employed by the attacker and defender at a specific time, respectively. Here, each component $A_{i}$ or $D_{j}(1\leq i\leq M,$ 1≤j≤N) is a Bernoulli random variable taking values 1 or 0, indicating whether the corresponding strategy is activated (1 for activated, 0 for not activated).

When selecting their respective strategies, the attacker and defender need to infer the true target network state X based on their observation vectors Y and Z, and then decide which strategies to adopt based on this inference. Therefore, from the overall system perspective, both A and D depend on the true state X of the target network. However, in an adversarial environment, neither party can accurately know the actual strategy used by the opponent. Each can only estimate the opponent’s strategy choice based on its own inference of the target state X, i.e., obtaining P(A|X) and P(D|X). For the defender, the uncertainty regarding the attacker’s strategy P(A|X) constitutes the second key uncertainty induced by perspective bias, which directly affects the judgment of the threat landscape during pre-engagement planning. The dynamic mechanism capturing multi-stage attack behaviors is detailed in Section 2.3.

**Figure 1:**
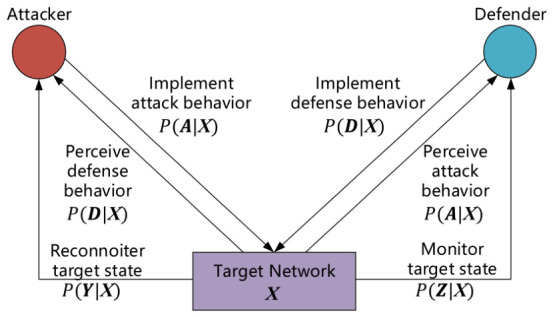
Relationships among the attacker, the defender, and the target network.

Figure 1 illustrates the relationships among the attacker, defender, and target network during one round of the cyber attack–defense process.

### 2.2. Uncertainty in State Evolution

In the attack–defense confrontation within ICS, the dynamic evolution of the system state is central to effectiveness evaluation. This framework attributes the uncertainty in state evolution to two main sources: uncertainty directly triggered by attack–defense actions, and uncertainty arising from dependency relationships among internal system devices. A key modeling choice underpinning this section is that the direct effects of attack–defense actions on a device’s state and the indirect effects propagated through inter-device dependencies are treated as probabilistically independent mechanisms. The following subsections develop each component in turn, and a detailed justification of this independence is provided in the Remark at the end of this section.

#### 2.2.1. State Evolution Uncertainty Triggered by Attack–Defense Actions

Attack and defense actions are the dominant factors driving rapid changes in the system state over the short term. The strategies employed by the attacker and defender succeed or fail with certain probabilities, thereby exerting random influences on the target system’s state. Let the target network state at time *t* be denoted by X(t), and the actions of the attacker and defender be represented by A(t) and D(t), respectively. When the target network is influenced by A(t) and D(t), its state transitions from X(t) at time *t* to X(t+1) at time t+1. Assuming the state transition possesses the Markov property, i.e., the next state depends only on the current state and the strategies of both parties, the state transition probability can be formally expressed as

(1) $$P\left(X(t+1)|X(0:t),A(0:t),D(0:t)\right)=P\left(X(t+1)|X(t),A(t),D(t)\right)$$

The conditional probability P(X(t+1)|X(t),A(t),D(t)) characterizes the influence and uncertainty of different attack–defense strategy combinations A(t),D(t) on the target state. For example, if the initial target state is $X\left(t\right)=x_{1}$, and the attacker can employ various strategies (e.g., $a,a^{\prime }$) to force the target into state $X\left(t+1\right)=x_{2}$, then the conditional probabilities $P(X\left(t+1\right)=x_{2}|X\left(t\right)=x_{1},A\left(t\right)=a)$ and $P(X\left(t+1\right)=x_{2}|X\left(t\right)=x_{1},$$A\left(t\right)=a^{\prime })$ reflect the success probabilities of attack strategies a and $a^{\prime }$, respectively.

**Remark on the Markov assumption in adversarial settings.** It is important to distinguish between the memoryless property of state transitions and the potentially history-dependent nature of attack and defense strategies. The Markov property formalized in Equation (1) applies exclusively to the evolution of the system state X(t). Its validity rests on the following observation: at time *t*, the distribution of X(t) already encodes all historical influences from the initial time through t−1, including prior attack actions, defense responses, and state degradations. Consequently, when conditioning on X(t) to predict X(t+1), the earlier trajectory X(0:t−1) provides no additional information about the next state. This reflects a modeling stance grounded in ICS risk assessment practice: for the discrete degradation levels employed (e.g., normal to complete failure per NIST SP 800-82 [20]), the device’s current physical condition serves as a sufficient proxy for its operational history, so the immediate effect of an action depends on that condition and the action itself, not on the specific path taken to reach it.

#### 2.2.2. State Evolution Uncertainty Induced by Dependency Relationships

Besides attack–defense actions, uncertainty in the system state also originates from functional dependencies among devices, as devices in the network do not operate in isolation; their states are interconnected through control commands, data flows, or physical couplings. A state change in one device may probabilistically affect, amplify, or suppress the states of other devices. This dependency-induced uncertainty is captured through conditional probabilities between device state variables, e.g., $P(X_{j}\left(t\right)|X_{i}\left(t\right))$ and $P(X_{j}\left(t+1\right)|X_{i}\left(t+1\right))$. We assume that the dependency relationships among devices remain stable during the evaluation period, i.e., $P\left(X_{j}\left(t\right)|X_{i}\left(t\right)\right)=P\left(X_{j}\left(t+1\right)|X_{i}\left(t+1\right)\right)$. This time-invariant assumption ensures the stability of the model structure, making it suitable for short-term confrontation analysis.

**Remark on the independence assumption.** For a given device, the direct effects of attack–defense actions on its state and the indirect effects propagated through inter-device dependencies are treated as probabilistically independent mechanisms. This independence is rooted in the distinct physical origins of the two influence sources: direct attack–defense effects depend on the technical characteristics of adversarial actions and defense responses, whereas dependency effects arise from the functional topology of the ICS. Because these two mechanisms operate through different causal pathways, treating them as conditionally independent is a principled structural decomposition: an attacker’s action exploits technical vulnerabilities of the target device (e.g., a buffer overflow in firewall firmware), whereas an upstream device failure alters the communication or power conditions of the target device (e.g., a switch outage disabling network connectivity). The outcome of one pathway does not directly influence the parameters governing the other. This decomposition also serves a pragmatic purpose: it allows the high-dimensional state transition CPT to be constructed from two lower-dimensional, more interpretable CPTs, rather than requiring the defender to directly specify a CPT over the full joint space of all parent nodes—a task that is combinatorially intractable. In scenarios where strong synergistic degradation effects exist, the bounded additive fusion rule employed in Section 2.5 provides a conservative approximation by capping the combined degradation at the maximum failure level.

### 2.3. Attack–Defense Model Based on DBN

#### 2.3.1. DBN Formulation

To model the uncertainty throughout the entire attack–defense confrontation process, this section introduces the DBN [21], integrating the core elements described previously—the target’s true state, observed states, and attack–defense strategies—along with their dynamic interrelationships into a temporal probabilistic framework.

**Figure 2:**
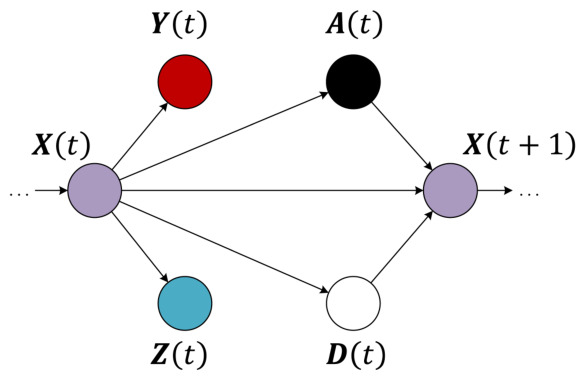
Dependencies among the observed state, true state, and attack/defense strategies.

A complete attack–defense confrontation process is discretized into multiple consecutive time slots. The sequence of the target network’s true states is denoted by (…,X(t),X(t+1),…); the sequences of observed states for the attacker and defender are (…,Y(t),Y(t+1),…) and (…,Z(t),Z(t+1),…), respectively. Based on this, a DBN is constructed as shown in Figure 2, where the dependencies between nodes are defined as follows:

**Observational Dependency:** The observations of the attacker and defender at time *t*, Y(t) and Z(t), are both determined by the target’s true state X(t) at that time.

**Strategic Dependency:** The strategy choices of both parties at time *t*, A(t) and D(t), are made independently based on their respective beliefs about the target network’s current true state X(t).

**State Evolution Dependency:** The target network’s state at time t+1, X(t+1), is jointly determined by its current state X(t) and the attack–defense actions A(t) and D(t) at that time.

This DBN model clearly reveals the transmission mechanism of dynamic attack–defense strategy interactions: the strategies A(t) and D(t) influence the target network’s state X(t+1) at time t+1, which in turn affects both parties’ observed states and subsequent strategy decisions A(t+1) and D(t+1) at time t+1, forming a closed-loop dynamic game process.

**Figure 3:**
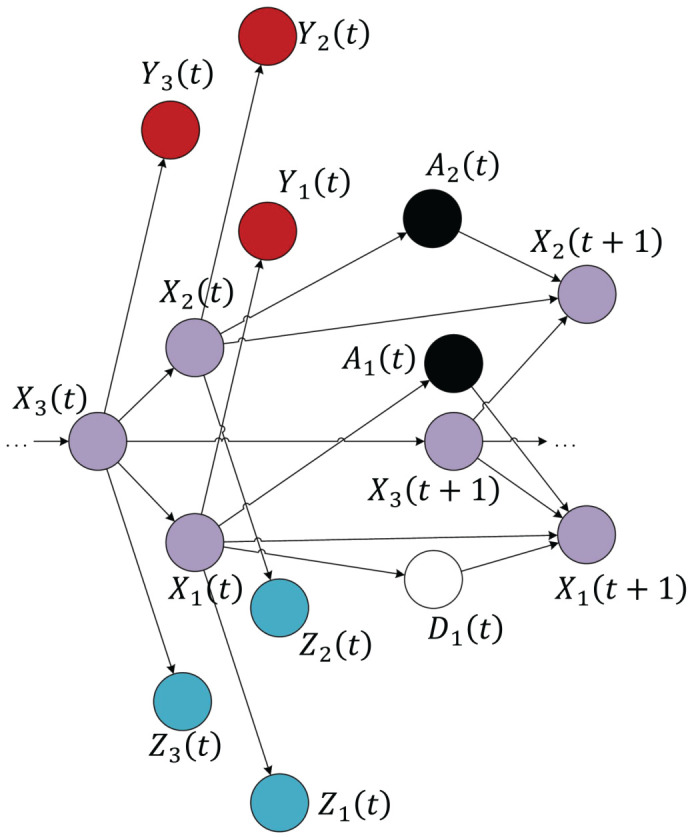
Example of dependencies between the observed state, true state, and attack/defense strategies (expanded view).

It is notable that internal dependencies exist between the components of the vectors X(t) and X(t+1) in Figure 2, determined by the inherent functional dependencies among devices within the target network. For a detailed illustration, Figure 3 shows an example of an expanded DBN where the vector components are explicitly represented. In this network, $X_{1}$ and $X_{2}$ both depend on $X_{3}$, reflecting the mutual influences between device states in real-world systems.

To facilitate concise expression in the subsequent text, we adopt the following notational convention: for any random variable *v* in the DBN, let par(v) denote the set of its parent nodes; for a set *V* of random variables, let $par\left(V\right)=\cup_{v\in V}par\left(v\right)$ denote the union of the parent node sets of all random variables in *V*. Unless otherwise specified, the terms “node” and its corresponding random variable are used interchangeably in the following discussion.

The DBN constructed in this section unifies the two categories of uncertainty—perspective bias and state evolution—within a temporally probabilistic framework with a rigorous mathematical foundation. This provides the core inference model for subsequent dynamic effectiveness evaluation from the defender’s perspective, oriented towards pre-engagement planning.

#### 2.3.2. DBN Modeling Choices

**On the dependency structure.** The directed acyclic graph encoding functional and logical dependencies among ICS devices may vary over time due to changes in network conditions or communication reachability. The DBN-based evaluation operates on a single time-step horizon: the inference from X(t) to X(t+1) requires only the dependency structures internal to each of these two time slices, which need not be identical. Because the evaluation is performed prior to engagement, the graph governing X(t+1) reflects the defender’s prior structural belief at time *t* rather than an observation-derived update from the future. The framework is designed to accept a different graph at each evaluation cycle as a self-contained input; while real-time structure learning from operational data lies outside the scope of this work, its output can be directly supplied as the dependency graph for any cycle. The fixed structure employed in our case study reflects the pre-engagement planning assumption that the system architecture is known to the defender over the short evaluation horizon, consistent with the operational context of ICS. This static treatment is deliberately scoped to single-step pre-engagement evaluation; continuous structural adaptation during long-horizon operation is not addressed in the present work.

**On robustness to dependency misspecification.** Even when the specified dependency graph deviates from reality, the framework exhibits a degree of resilience. The CPT perturbation analysis presented later in this work can be interpreted as a stress test for this robustness: by injecting noise into the conditional probability parameters that encode the dependency strengths among device states—i.e., the terms $P(X_{j}\left(t\right)|X_{i}\left(t\right))$ that, together with the marginal priors, factorize the joint prior distribution P(X(t)) according to the dependency graph—we examine whether the dual-metric evaluation remains informative under moderate levels of parameter misspecification. This design provides a principled means to assess the framework’s reliance on accurate dependency specifications.

**On encoding attack memory through state mediation.** As noted in Section 2.1.2, the attacker’s strategy A(t) depends solely on the current system state X(t), this does not imply that the attacker is memoryless. Multi-stage attack behaviors are encoded through the state-mediated path A(t)→X(t+1)→A(t+1): each action alters the system state, and the attacker’s subsequent decision—while formally conditioned only on X(t+1)—is implicitly informed by the entire attack chain because the posterior belief over X(t+1) encodes the consequences of all preceding observations and actions.

This structure reflects a standard modeling stance in game-theoretic security modeling: the defender treats the attacker as a rational agent whose strategy choice responds to the current game state X(t). Because X(t) is shaped by all prior attack and defense interactions, the state-conditioned decision rule P(A(t)∣X(t)) provides a compact representation of history-dependent behavior without introducing direct temporal links between successive strategy nodes. The defender does not need to know the attacker’s true decision rule; rather, the defender specifies a belief over possible attacker strategies, represented by the conditional distribution P(A(t)∣X(t)) in the model.

### 2.4. Metrics of Attack–Defense Effectiveness

The effectiveness of a target network refers to its capability to achieve predefined functions or missions when in a specific state, which is determined by its state X. To quantify this capability, the overall system effectiveness value *F* is defined as a function of the true state vector X:

$$\forall X\in \Omega_{X},\;F=\varphi \left(X\right).$$

Here, $\varphi \left(X\right):\Omega_{X}\to R$ is a mapping from the state space to the real number space, termed the effectiveness function.

The core impact of attack–defense actions is reflected in their driving effect on system state changes, which consequently alters system effectiveness. When the system state transitions from X(t) at time *t* to X(t+1) at time t+1, the corresponding change in effectiveness, namely the effectiveness differential ΔF, is defined as

ΔF=φ(X(t+1))−φ(X(t)).

This effectiveness differential directly characterizes the combined effect of the actions taken by both attacker and defender. Given the stochastic nature of state evolution, ΔF is a random variable. Its probability distribution comprehensively captures all possible outcomes of the attack–defense effect and can be calculated by

(2) $$P\left(\Delta F=\delta \right)=\sum_{\begin{array}{c} x,\;x^{\prime }\in \Omega_{X}\\ \varphi \left(x^{\prime }\right)-\varphi \left(x\right)=\delta \end{array}}P\left(X\left(t\right)=x,\;X\left(t+1\right)=x^{\prime }\right).$$

In Equation (2), the summation runs over all state pairs $\left(x,x^{\prime }\right)$ whose effectiveness difference equals δ. This reflects a two-stage process: the DBN inference yields the joint probability of each state transition, and the effectiveness function φ then maps each transition to a scalar effectiveness change. The probability of a given δ is thus the total probability mass of all transitions that produce that particular effectiveness differential.

Based on this probability distribution, we employ two core metrics to measure the average effect of attack–defense actions and the uncertainty of the outcome, respectively:

$$\begin{array}{cc} E(\Delta F) & =\sum_{\delta \in R}P\left(\Delta F=\delta \right)\cdot \delta ,\\ H(\Delta F) & =-\sum_{\delta \in R}P\left(\Delta F=\delta \right)\cdot \log P\left(\Delta F=\delta \right). \end{array}$$

Although the distribution of ΔF is defined in Equation (2) in its unconditional form, the defender’s evaluation always conditions on the available observation and chosen strategy. Accordingly, in the computational procedure, the metrics are computed from the posterior distribution P(ΔF=δ∣Z(t),D(t)), and the notation E(ΔF) and H(ΔF) in the remainder of this paper refer to these posterior quantities. In this context, H(ΔF) serves as a scalar measure of the total prediction uncertainty. All sources of uncertainty in the DBN framework are first structurally encoded into the posterior joint distribution P(X(t),X(t+1)∣Z(t),D(t)), from which the distribution of ΔF is obtained via Equation (2). The entropy metric thus reflects the total prediction uncertainty without having to disentangle its sources, since the structural separation of distinct uncertainty types is already accomplished by the DBN. The relationship between entropy and alternative measures such as variance is discussed in Section 4.3.

From the defender’s perspective, an ideal defense strategy should yield a high positive E(ΔF) and a low H(ΔF), i.e., a high expected return with stable, predictable outcomes. When the expected gains are similar, the defender should prioritize strategies with lower entropy to enhance decision robustness.

**Remark on the role of the effectiveness function.** The effectiveness function φ(X) serves as a controlled input that formalizes the defender’s organizational priorities and risk appetite. It is not a model assumption to be validated against empirical data, but rather a value judgment that maps system states to a scalar measure of operational effectiveness. The framework is designed to accept any suitably defined effectiveness function and does not depend on the particular weights or contribution values chosen. It is important to note, therefore, that the focus of this work is not on how the state vector X is selected or the specific form of φ(X), but rather on how to quantify the change in system effectiveness induced by attack–defense actions and its associated uncertainty, once these elements are defined.

### 2.5. Computation of Attack–Defense Effectiveness

After establishing the attack–defense model and the effectiveness metrics, this section addresses the core problem: how can the defender, in practice, compute the effectiveness expectation and uncertainty for different strategies. Specifically, the defender aims to evaluate the effect of employing a specific defense strategy D(t) based on its observed state Z(t) at time *t*.

The following provides a formal description of the effectiveness computation problem from the defender’s perspective, followed by a breakdown of the computational procedure to elucidate its mathematical essence and required parameters.

**Problem Description (Defender’s Perspective):** Based on the probabilistic relationships captured by the DBN, the defender, given its observation Z(t)=z at time *t*, computes the probability distribution P(ΔF=δ∣Z(t)=z,D(t)=d) of the effectiveness differential for the target network from time *t* to t+1, given the adoption of defense strategy D(t)=d. Subsequently, it computes the expectation E(ΔF∣Z(t)=z,D(t)=d) and the information entropy H(ΔF∣Z(t)=z,D(t)=d).

Note that the inference relies exclusively on the defender’s observation Z(t) and the prior over system states; the attacker’s observation vector Y(t), while defined in the full DBN structure for modeling completeness, is immaterial to the defender’s computational workflow and does not enter any of the subsequent equations.

#### 2.5.1. Theoretical Computational Procedure

According to the definition of ΔF, we have

$$\begin{array}{cc} \; & P(\Delta F=\delta |Z(t)=z,D(t)=d)\\ \; & =\sum_{\begin{array}{c} x,\;x^{\prime }\in \Omega_{X}\\ \varphi \left(x^{\prime }\right)-\varphi \left(x\right)=\delta \end{array}}P\left(X\left(t\right)=x,X\left(t+1\right)=x^{\prime }|Z\left(t\right)=z,D\left(t\right)=d\right). \end{array}$$

Therefore, computing the probability distribution of the effectiveness differential is equivalent to computing the posterior joint probability distribution of X(t) and X(t+1):

$$\begin{array}{cc} \; & P(X\left(t\right)=x,X\left(t+1\right)=x^{\prime }|Z\left(t\right)=z,D\left(t\right)=d)\\ \; & =P\left(X\left(t+1\right)=x^{\prime }|X\left(t\right)=x,D\left(t\right)=d\right)\cdot P\left(X\left(t\right)=x|Z\left(t\right)=z\right). \end{array}$$

This can be further decomposed into two steps:

**Step 1.** Infer the probability distribution of the target network’s true state based on the observed state (reverse inference), computing P(X(t)=x∣Z(t)=z):

$$P\left(X\left(t\right)=x|Z\left(t\right)=z\right)=\frac{P(Z(t)=z|X(t)=x)P(X(t)=x)}{\sum_{x^{\prime }\in \Omega_{X}}P\left(Z\left(t\right)=z|X\left(t\right)=x^{\prime }\right)P\left(X\left(t\right)=x^{\prime }\right)}.$$

This step requires the following inputs:

($I_{1}$) P(X(t)=x): the prior distribution of X(t).
($I_{2}$) P(Z(t)=z∣X(t)=x): the likelihood probability of the observed state given the true state in the state observation model.

Regarding ($I_{1}$) and ($I_{2}$), the chain rule can be applied to further expand X(t) and Z(t):

$$\begin{array}{cc} P(X(t)) & =\prod_{i=1}^{I}P\left(X_{i}\left(t\right)|par\left(X_{i}\left(t\right)\right)\right),\\ P(Z(t)|X(t)) & =\prod_{k=1}^{K}P\left(Z_{k}\left(t\right)|par\left(Z_{k}\left(t\right)\right)\right). \end{array}$$

In the above equations, if $par(X_{i}\left(t\right))=⌀$, then $P\left(X_{i}\left(t\right)|par\left(X_{i}\left(t\right)\right)\right)=P\left(X_{i}\left(t\right)\right)$ is the prior probability. Because the random variables in Z(t) are conditionally independent given X(t), we have $par(Z_{k}\left(t\right))\subseteq X\left(t\right)$.

**Step 2.** Predict the probability distribution of the state at the next time step based on the current state and attack strategies, computing $P(X\left(t+1\right)=x^{\prime }|X\left(t\right)=x,D\left(t\right)=d)$:

(3) $$\begin{array}{cc} \; & P(X\left(t+1\right)=x^{\prime }|X\left(t\right)=x,D\left(t\right)=d)\\ \; & =\sum_{a}P\left(X\left(t+1\right)=x^{\prime },A\left(t\right)=a|X\left(t\right)=x,D\left(t\right)=d\right)\\ \; & =\sum_{a}P\left(A\left(t\right)=a|X\left(t\right)=x,D\left(t\right)=d\right)\cdot P\left(X\left(t+1\right)=x^{\prime }|X\left(t\right)=x,A\left(t\right)=a,D\left(t\right)=d\right)\\ \; & =\sum_{a}P\left(A\left(t\right)=a|X\left(t\right)=x\right)\cdot P\left(X\left(t+1\right)=x^{\prime }|X\left(t\right)=x,A\left(t\right)=a,D\left(t\right)=d\right). \end{array}$$

This step requires the following inputs:

($I_{3}$) P(A(t)=a∣X(t)=x): the decision strategy of the attacker.
($I_{4}$) $P(X\left(t+1\right)=x^{\prime }|X\left(t\right)=x,A\left(t\right)=a,D\left(t\right)=d)$: the state transition probability triggered by attack–defense actions.

Regarding ($I_{3}$) and ($I_{4}$), similarly expanding A(t) and X(t+1) using the chain rule yields

$$\begin{array}{cc} P(A(t)|X(t)) & =\prod_{i=1}^{M}P\left(A_{i}\left(t\right)|par\left(A_{i}\left(t\right)\right)\right),\\ P(X(t+1)|X(t),A(t),D(t)) & =\prod_{i=1}^{I}P\left(X_{i}\left(t+1\right)|par\left(X_{i}\left(t+1\right)\right)\right). \end{array}$$

If correlations between different attack strategies are not considered, then $par(A_{i}\left(t\right))\subseteq X\left(t\right)$.

A key challenge in constructing the DBN is that directly defining the high-dimensional conditional probability $P(X_{i}\left(t+1\right)|par\left(X_{i}\left(t+1\right)\right))$ is often infeasible. For a device with *k* parent nodes each taking *m* possible states, the full CPT requires $m^{k+1}$ entries, which quickly becomes intractable for manual specification or expert elicitation. To address this, we adopt a decomposition and fusion approach based on a noise model [21].

Specifically, we partition the parent node set $par(X_{i}\left(t+1\right))$ into *L* groups according to the physical nature of their influence, denoted $par_{1},par_{2},\ldots ,par_{L}$, such that ${⋃}_{l=1}^{L}par_{l}=par\left(X_{i}\left(t+1\right)\right)$. The core assumption is that, given the child node $X_{i}\left(t+1\right)$, the influences from different parent groups are conditionally independent. Under this assumption, we first define a separate base conditional probability table $P(X_{i}\left(t+1\right)|par_{l})$ for each group $par_{l}$, each of which has substantially lower dimensionality than the full CPT. Subsequently, a formal superposition rule is applied to fuse these independent base CPTs into the complete CPT $P(X_{i}\left(t+1\right)|par\left(X_{i}\left(t+1\right)\right))$.

The superposition rule adopted in this work is a bounded additive model: if, under the influence of two independent parent groups, the state of device $X_{i}$ would be driven to degradation levels $s_{a}$ and $s_{b}$ respectively, then under their combined influence the resulting state is defined as $\min\{s_{\max},s_{a}+s_{b}\}$, where $s_{\max}$ denotes the maximum possible degradation level (i.e., complete failure). This rule reflects the physical intuition that simultaneous stress factors produce cumulative degradation, but with saturation at the complete failure state. The fusion of more than two parent groups proceeds by sequential pairwise application of this rule.

Taking node $X_{1}\left(t+1\right)$ in Figure 3 as an example, its parent nodes are $\left\{X_{3}\left(t+1\right),X_{1}\left(t\right),A_{1}\left(t\right),D_{1}\left(t\right)\right\}$. These can be partitioned into two independent groups:

- $par_{1}=\left\{X_{3}\left(t+1\right)\right\}$, whose base CPT $P(X_{1}\left(t+1\right)|X_{3}\left(t+1\right))$ characterizes the structural dependency influence from other devices in the network;
- $par_{2}=\left\{X_{1}\left(t\right),A_{1}\left(t\right),D_{1}\left(t\right)\right\}$, whose base CPT $P(X_{1}\left(t+1\right)|X_{1}\left(t\right),A_{1}\left(t\right),D_{1}\left(t\right))$ characterizes the state transition driven by the device’s own current state and the attack–defense actions.

The complete CPT for $X_{1}\left(t+1\right)$ is then generated by fusing $P(X_{1}\left(t+1\right)|X_{3}\left(t+1\right))$ and $P(X_{1}\left(t+1\right)|X_{1}\left(t\right),A_{1}\left(t\right),D_{1}\left(t\right))$ using the bounded additive superposition rule. This decomposition transforms the complex global probability assessment into multiple simpler, locally interpretable assessments with clear physical meanings, significantly enhancing the feasibility and interpretability of model construction.

#### 2.5.2. Practical Implementation

In practical implementation, the posterior joint distribution P(X(t),X(t+1)∣Z(t),D(t)) is obtained through standard DBN inference. Converting this distribution into the distribution of the effectiveness differential ΔF via Equation (2) requires mapping each state pair $\left(x,x^{\prime }\right)$ to a scalar effectiveness difference $\delta =\varphi \left(x^{\prime }\right)-\varphi \left(x\right)$.

A direct enumeration over all $|\Omega_{X}{|}^{2}$ state pairs, as suggested by the summation in Equation (2), would be computationally prohibitive and memory-intensive for large state spaces. This bottleneck is eliminated through a two-stage memory-optimized strategy:

**Offline precomputation.** A one-dimensional array eff[x]=φ(x) is precomputed for all $x\in \Omega_{X}$. This array stores the total system effectiveness for each single-time-step state configuration, requiring only $O(|\Omega_{X}|)$ storage and computation time.

**Online evaluation.** The posterior joint distribution produced by DBN inference is typically highly sparse due to the conditional independencies encoded in the network structure. Rather than iterating over all $|\Omega_{X}{|}^{2}$ entries, only those state pairs with non-negligible probability mass are retained; for each such pair, IT computes $\delta =\mathrm{eff}\left[x^{\prime }\right]-\mathrm{eff}\left[x\right]$ via two array lookups and one subtraction. The probability mass is then accumulated into the corresponding bin of P(ΔF=δ). The computational cost of this operation scales linearly with the number of non-zero probability entries rather than with the size of the full state space.

This offline–online separation is a direct consequence of the framework’s modular architecture: the effectiveness function φ is a controlled input decoupled from the DBN structure (see the Remark at the end of Section 2.4), allowing the mapping to be computed once and reused universally. The DBN inference itself is a standard task whose computational complexity is inherent to all DBN-based methods and is not specific to this framework; mature inference algorithms and optimized toolkits such as pgmpy [22] are available for this purpose.

### 2.6. Summary of Notation

For the reader’s convenience, Table 1 provides a consolidated summary of the key symbols introduced throughout Section 2, grouped by their functional roles in the framework.

## 3. Results

### 3.1. Network Scenario Design

**Figure 4:**
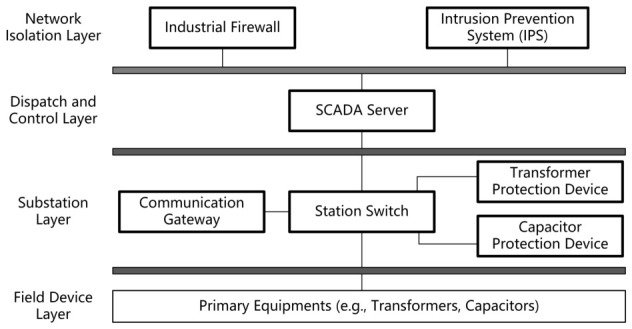
Network topology of the substation automation system.

To demonstrate the capability of the proposed dynamic evaluation framework in assessing defense strategies and quantifying prediction uncertainty, a typical substation automation system is analyzed as the target network. This network adheres to the security principle of “hierarchical partitioning and longitudinal encryption” for electric power systems. The network topology, as shown in Figure 4, is primarily divided into four logical layers: the Network Isolation Layer, the Dispatch and Control Layer, the Substation Layer, and the Field Device Layer. The network devices contained within each layer are described below.

#### 3.1.1. Network Isolation Layer

This layer constitutes the boundary between the entire system and external networks. It implements access control, intrusion detection, and security domain isolation through the deployment of a series of specialized security devices. Its core assets include the following:

Industrial Firewall:Executes fine-grained access control policies, permitting only authorized communication protocols (e.g., IEC 60870-5-104 [23], secured in accordance with IEC 62351-3 [24]) and data flows.

Intrusion Detection System (IDS): Performs deep inspection of network traffic crossing this boundary, identifying and blocking malicious attacks (e.g., DDoS attacks, vulnerability exploitation) in real-time, effectively suppressing the penetration of threats into the internal network.

This layer is a critical component reflecting the defender’s information advantage and the implementation of proactive defense strategies. The effectiveness of its policies directly impacts the reliability and security of external communications for devices in the Dispatch and Control Layer and Substation Layer.

#### 3.1.2. Dispatch and Control Layer

This layer serves as the control core of the entire system, deployed within a physically secure and logically isolated internal private network. Its main function is the centralized monitoring, data management, and dispatch decision-making for all substation facilities. Its core asset includes:

SCADA Server: Acting as the “brain” of the system, it is responsible for collecting real-time operational data (e.g., voltage, current, circuit breaker status) from the entire network, performing monitoring and data parsing, and sending control commands to lower-level units.

#### 3.1.3. Substation Layer

This layer acts as a regional power distribution hub, responsible for aggregating data from field devices within its jurisdiction and executing control commands from upper-level units. Its core assets include the following:

Station Switch: This forms the local area network within the substation, connecting all station-level and device-level equipment.

Communication Gateway: This is responsible for communicating with remote dispatch centers or other systems, converting different communication protocols.

Protection Devices: Examples include Transformer Protection and Capacitor Protection Devices, which are the core of the relay protection system, responsible for quickly isolating faulty equipment to ensure power grid safety.

#### 3.1.4. Field Device Layer

This layer includes primary equipment such as transformers and capacitors, which represent the core execution level that directly interacts with the high-voltage equipment of the power system. Their operational status directly determines the realization of the grid’s physical functions. Due to the strong functional coupling between protection devices and primary equipment, the status of a protection device can indirectly reflect the operational safety and protection level of the corresponding primary equipment. Furthermore, data from protection devices is more readily obtainable through the SCADA Server or maintenance logs. Therefore, in the Bayesian network modeling for this case study, primary equipment is not directly included as nodes. Instead, the focus is solely on their corresponding protection devices (e.g., Transformer Protection Device, Capacitor Protection Device). This approach simplifies the network structure while ensuring assessment accuracy, thereby improving computational efficiency in pre-engagement planning context.

### 3.2. Parameter Settings

Based on the parameter acquisition principles outlined in Section 4.4, this case study employs simulated methods to generate the required CPTs to validate the core framework’s effectiveness. All simulated data can be accessed via [25].

#### 3.2.1. State Vectors and Dependency Relationships

This case study constructs the DBN based on the states of the following nodes: Industrial Firewall ($X_{1}$), IDS ($X_{2}$), SCADA Server ($X_{3}$), Station Switch ($X_{4}$), Communication Gateway ($X_{5}$), Transformer Protection Device ($X_{6}$), and Capacitor Protection Device ($X_{7}$), to evaluate the effectiveness of the target network. Referencing the severity classification logic for ICS security events in NIST SP 800-82 [20] and considering the failure modes inflicted on devices by actual attack–defense scenarios, the operational state of each device is divided into four levels (e.g., normal, minor degradation, severe degradation, complete failure), identified by $X_{i}=0,1,2,3\left(i=1,2,\ldots ,7\right)$, covering typical scenarios from performance degradation to complete failure.

**Figure 5:**
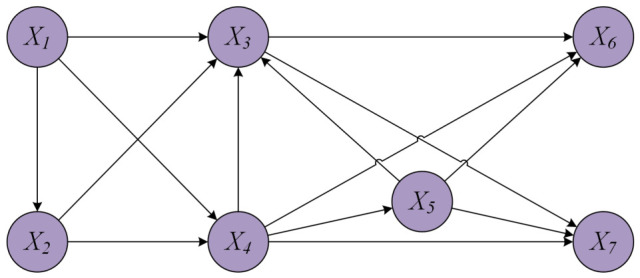
Dependency relationships among the states of various devices.

When constructing the DBN model characterizing the states of these devices, the dependency relationships between nodes in the network are defined as a comprehensive representation of existing functional influence relationships, control/command dependencies, and communication reachability dependencies. Specifically, (1) Functional Influence Dependency exists if a failure or performance degradation of Device A directly leads to functional limitations, abnormalities, or failures in Device B. Such dependencies reflect the direct influence relationships between devices in terms of physical operation, logical function, or security protection within the system. (2) Control/Command Dependency exists if the operational state, control logic, or configuration parameters of Device B are issued, triggered, or adjusted by Device A. This dependency embodies the chain of command transmission and control action from upper-level control devices to lower-level execution devices in the system. (3) Communication Reachability Dependency exists if the communication status, network configuration, or protocol conversion capability of Device A affects the information exchange, monitoring, or control data transmission between Device B and other devices. This dependency reflects the reachability constraints in the information channels and data flows of the system. In summary, if a failure, misconfiguration, or communication anomaly in Device A cause degradation in the functionality, control, or communication capability of Device B, a directed edge from A to B is established in the DBN to characterize this probabilistic dependency. During topology construction, only direct dependencies are considered, ensuring the network structure remains a directed acyclic graph (DAG) to guarantee the correctness of causal inference and probability propagation. Based on this, the Bayesian network shown in Figure 5 is used to characterize the correlations between the state variables $X_{i}\left(i=1,2,\ldots ,7\right)$ (nodes represent random variables, edges represent dependencies between random variables):

The dependency relationships among the nodes are summarized as follows:

1. The firewall, located at the outermost security boundary of the system, has its policies independently configured and actively enforces access control. It does not depend on the operational state of any other network or functional node.
2. The IDS depends on the firewall. This is because the firewall determines the scope and security policy of allowed traffic. Its configuration or failure directly affects the volume and content of network traffic received by the IDS, thereby influencing its detection load and analysis results.
3. The operation of the SCADA Server depends on the firewall, IDS, gateway, and Station Switch. The Firewall and IDS together form the outer protective boundary of the SCADA Server’s network. Abnormal operation of either the firewall or IDS may expose the SCADA Server to security threats, affecting its normal operation. The gateway handles cross-domain communication and protocol conversion, while the Station Switch provides the fundamental communication channel for the SCADA Server to interact with station devices (e.g., protection devices). Malfunctions in either the gateway or the switch would interrupt the SCADA Server’s data acquisition and control command transmission.
4. The Communication Gateway depends on the Station Switch. The Station Switch provides the gateway with the intra-station data forwarding path and uplink channel. If the switch fails, the gateway will be unable to communicate with other devices, causing its protocol conversion and data relay functions to fail.
5. The Station Switch, as the core communication device within the substation, has fewer upstream dependencies and primarily provides basic connectivity functions.
6. Various protection devices collectively depend on the SCADA Server, Communication Gateway, and Station Switch. The switch and gateway provide them with communication and relay capabilities, while the SCADA Server issues operational parameters and control commands to manage them. Failures or anomalies in the switch, gateway, or SCADA Server will constrain the monitoring, control, or configuration functions of the protection devices, thereby affecting their protective actions and logic judgments.

In summary, for this case study, the prior distribution P(X(t)=x) is

$$\begin{array}{cc} P( & X\left(t\right)=x)=P\left(X_{1}\left(t\right),X_{2}\left(t\right),X_{3}\left(t\right),X_{4}\left(t\right),X_{5}\left(t\right),X_{6}\left(t\right),X_{7}\left(t\right)\right)\\ \; & =P\left(X_{1}\left(t\right)\right)\cdot P\left(X_{2}\left(t\right)|X_{1}\left(t\right)\right)\cdot P\left(X_{3}\left(t\right)|X_{1}\left(t\right),X_{2}\left(t\right),X_{4}\left(t\right),X_{5}\left(t\right)\right)\\ \; & \;\cdot P\left(X_{4}\left(t\right)|X_{1}\left(t\right),X_{2}\left(t\right)\right)\cdot P\left(X_{5}\left(t\right)|X_{4}\left(t\right)\right)\\ \; & \;\cdot P\left(X_{6}\left(t\right)|X_{3}\left(t\right),X_{4}\left(t\right),X_{5}\left(t\right)\right)\cdot P\left(X_{7}\left(t\right)|X_{3}\left(t\right),X_{4}\left(t\right),X_{5}\left(t\right)\right). \end{array}$$

Before conducting effectiveness evaluation, both attackers and defenders must pre-obtain the 7 CPTs related to $X_{i}\left(t\right)\left(i=1,2,\ldots ,7\right)$.

#### 3.2.2. Observation Vectors

The defender’s observation vector is defined as $Z=(Z_{1},Z_{2},\ldots ,Z_{7})$, where $Z_{i}$ corresponds to $X_{i}$ (i=1,2,…,7), but potential errors exist, characterized by $P(Z_{i}\left(t\right)|X_{i}\left(t\right))$. This case study assumes the defender has a low observation error rate, with a misjudgment rate constrained to <5%; the detection rate for the “complete failure” state is 100%.

#### 3.2.3. Attack–Defense Strategies and State Transition Model

To facilitate model computation and reasoning analysis, we define one typical attack strategy and one corresponding defense strategy for each of the five key devices, Firewall, IDS, SCADA Server, Station Switch, and Communication Gateway, based on the aforementioned network structure. The strategy vectors for the attacker and defender are denoted as $A=\{A_{1},A_{2},\ldots ,A_{5}\}$ and $D=\{D_{1},D_{2},\ldots ,D_{5}\}$, respectively, where each component’s value indicates whether the strategy is executed. The combination of these strategies at time slot *t* collectively determines the evolution of the system state vector X(t) to X(t+1).

- $A_{1}$: The attacker modifies firewall policies through credential leakage or vulnerability exploitation, relaxing access control and opening sensitive ports, allowing unauthorized traffic into the internal network.
- $D_{1}$: The defender prevents unauthorized firewall modification through the principle of least-privilege and two-factor authentication.
- $A_{2}$: The attacker evades IDS detection using packet fragmentation, encrypted tunnels, or signature mutation, allowing malicious traffic to go undetected.
- $D_{2}$: The defender employs a combined rule-based and behavioral detection mechanism, regularly updating signature libraries to improve the identification rate of evasive traffic.
- $A_{3}$: The attacker injects malicious code exploiting system vulnerabilities to tamper with monitoring data or execute incorrect control commands, causing SCADA functional anomalies or misoperations.
- $D_{3}$: The defender prevents malicious code injection through system hardening, timely patching, and application signature verification, restoring normal SCADA logic.
- $A_{4}$: The attacker exhausts switch resources by sending a large volume of spoofed broadcast or abnormal packets, causing LAN congestion or outage.
- $D_{4}$: The defender mitigates flooding and abnormal traffic attacks by enabling MAC address binding, port security, and QoS rate limiting.
- $A_{5}$: The attacker intercepts and tampers with control or measurement data in the inter-station communication channel, compromising protocol integrity and communication trust.
- $D_{5}$: The defender prevents man-in-the-middle tampering of communications by enabling encrypted channels (TLS/IEC 62351) and message authentication codes.

For any attack strategy $A_{i}$ or defense strategy $D_{j}$ (j=1,2,…,5), $X_{i}$ is the device state affected by strategies $A_{i}$ and $D_{i}$. The implementation of strategies by attackers and defenders depends only on the true state of the target network. Therefore, in this case study, $P\left(D\left(t\right)|X\left(t\right)\right)=\prod_{i=1}^{5}P\left(D_{i}\left(t\right)|X_{i}\left(t\right)\right)$ and $P\left(A\left(t\right)|X\left(t\right)\right)=\prod_{i=1}^{5}P\left(A_{i}\left(t\right)|X_{i}\left(t\right)\right)$. When simulating P(D(t)∣X(t)) and P(A(t)∣X(t)), we adhere to the following principles:

1. Both attackers and defenders dynamically adjust the usage probability of their strategies based on the applicable scenarios and device states.
2. The attacker tends towards low-risk probing for normal devices and high-gain attacks for degraded devices.
3. The defender prioritizes deploying targeted measures for abnormal devices and reduces ineffective protection for failed devices.

In this case study, according to the correspondence between attack–defense strategies and devices, P(X(t+1)∣X(t),A(t),D(t)) can be expanded as follows:

$$\begin{array}{cc} P( & X(t+1)|X(t),A(t),D(t))\\ \; & =P(X_{1}\left(t+1\right)|X_{1}\left(t\right),A_{1}\left(t\right),D_{1}\left(t\right))\\ \; & \;\times P(X_{2}\left(t+1\right)|X_{1}\left(t+1\right),X_{2}\left(t\right),A_{2}\left(t\right),D_{2}\left(t\right))\\ \; & \;\times P(X_{3}\left(t+1\right)|X_{1}\left(t+1\right),X_{2}\left(t+1\right),X_{4}\left(t+1\right),X_{5}\left(t+1\right),X_{3}\left(t\right),A_{3}\left(t\right),D_{3}\left(t\right))\\ \; & \;\times P(X_{4}\left(t+1\right)|X_{1}\left(t+1\right),X_{2}\left(t+1\right),X_{4}\left(t\right),A_{4}\left(t\right),D_{4}\left(t\right))\\ \; & \;\times P(X_{5}\left(t+1\right)|X_{4}\left(t+1\right),X_{5}\left(t\right),A_{5}\left(t\right),D_{5}\left(t\right))\\ \; & \;\times P(X_{6}\left(t+1\right)|X_{3}\left(t+1\right),X_{4}\left(t+1\right),X_{5}\left(t+1\right),X_{6}\left(t\right))\\ \; & \;\times P(X_{7}\left(t+1\right)|X_{3}\left(t+1\right),X_{4}\left(t+1\right),X_{5}\left(t+1\right),X_{7}\left(t\right)). \end{array}$$

For any device, its state at time t+1 is influenced by two aspects: first, its own state at time *t* and the attack–defense actions it suffers; second, the states of other devices at time t+1. Furthermore, these two influences are assumed to be independent. Therefore, two types of conditional probabilities are generated for each $X_{i}\left(t+1\right)$: one reflecting the influence of its own state and attack–defense strategies at time *t*, and the other reflecting the correlations between devices. The conditional probabilities reflecting inter-device influences are kept consistent with the settings in P(X(t)=x). Taking $X_{2}\left(t+1\right)$ as an example, it requires two CPTs: $P(X_{2}\left(t+1\right)|X_{2}\left(t\right),A_{2}\left(t\right),D_{2}\left(t\right))$ and $P(X_{2}\left(t+1\right)|X_{1}\left(t+1\right))$, where $P\left(X_{2}\left(t+1\right)|X_{1}\left(t+1\right)\right)=P\left(X_{2}\left(t\right)|X_{1}\left(t\right)\right)$. For the state transition probabilities driven by the five pairs of attack–defense strategies, $P(X_{i}\left(t+1\right)|X_{i}\left(t\right),A_{i}\left(t\right),D_{i}\left(t\right))\left(i=1,2,\ldots ,5\right)$, we adhere to the following three principles:

1. When neither the attack nor the corresponding defense behavior is present, the device’s next state is always consistent with its current state:
  $$P\left(X_{i}\left(t+1\right)=x^{\prime }|X_{i}\left(t\right)=x,A_{i}=0,D_{i}=0\right)=\delta \left(x^{\prime }-x\right),$$
2. When only the attack exists without the corresponding defense, the device’s state cannot improve, only worsen:
  $$P\left(X_{i}\left(t+1\right)=x^{\prime }|X_{i}\left(t\right)=x,A_{i}=1,D_{i}=0\right)=0,\;\mathrm{if}\;x^{\prime }<x$$
3. When only the defense exists without the corresponding attack, the device’s state cannot degrade, only improve:
  $$P\left(X_{i}\left(t+1\right)=x^{\prime }|X_{i}\left(t\right)=x,A_{i}=0,D_{i}=1\right)=0,\;\mathrm{if}\;x^{\prime }>x$$

When fusing the influences of multiple factors on a device’s state at the next time step, we employ a bounded additive model: if, under the respective influence of two factors, the device’s potential new states are *x* and $x^{\prime }$ ($x,x^{\prime }\in \left\{0,1,2,3\right\}$), then the device’s state under the combined influence of both factors is assumed to be $\min\{3,x+x^{\prime }\}$. For example,

$$\begin{array}{cc} \; & P(X_{2}\left(t+1\right)=x_{2}^{\prime }|X_{1}\left(t+1\right)=x_{1},X_{2}\left(t\right)=x_{2},A_{2}\left(t\right)=a_{2},D_{2}\left(t\right)=d_{2})\\ \; & =\sum_{\min\left\{3,x_{2,1}^{\prime }+x_{2,2}^{\prime }\right\}=x_{2}^{\prime }}[P\left(X_{2}\left(t+1\right)=x_{2,2}^{\prime }|X_{1}\left(t+1\right)=x_{1}\right)\\ \; & \;\;\cdot P(X_{2}\left(t+1\right)=x_{2,1}^{\prime }|X_{2}\left(t\right)=x_{2},A_{2}\left(t\right)=a_{2},D_{2}\left(t\right)=d_{2})] \end{array}$$

#### 3.2.4. Complete DBN

**Figure 6:**
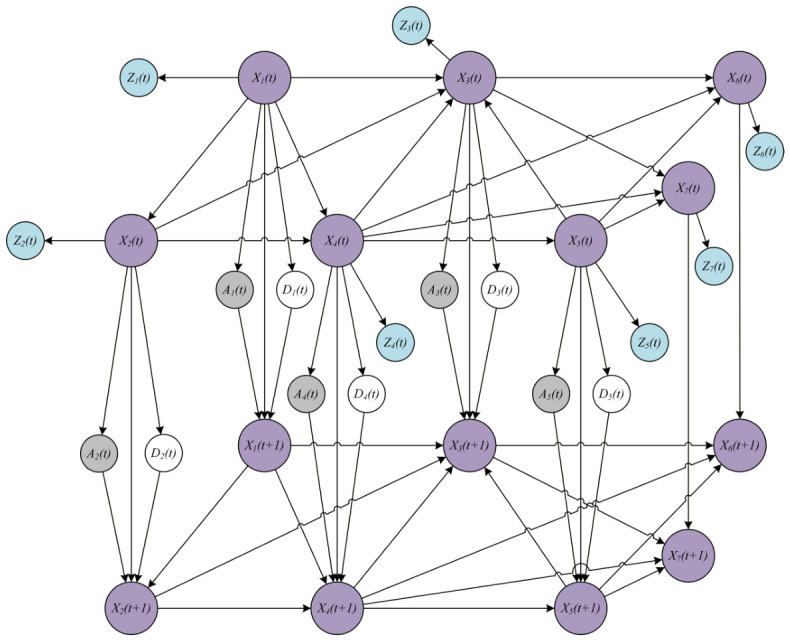
DBN for effectiveness evaluation of the substation automation system.

Based on the aforementioned dependency relationships among devices and the modeling of attack–defense behaviors, a complete DBN covering the entire process of system operation, observation, and decision-making is constructed from the defender’s perspective for this case study, as shown in Figure 6.

### 3.3. Effectiveness Function Definition

The state space $\Omega_{X}$ of the target network contains $4^{7}=16,384$ states. Based on these states, the overall system effectiveness value is defined as

$$F=\varphi \left(X\right)=\sum_{i=1}^{7}w_{i}\cdot \varphi_{i}\left(X_{i}\right)$$

where $w_{i}$ is the weight of each device, satisfying $\sum_{i=1}^{7}w_{i}=1$, and $\varphi_{i}\left(X_{i}\right)$ is the contribution of each device to the overall effectiveness at different states.

To model the functional differences and importance of the seven key devices within the system’s overall operation, we use a hierarchical weight allocation strategy. The SCADA Server, which undertakes global monitoring and dispatch decision-making functions whose failure directly leads to system-level control loss, is assigned the highest weight $w_{3}=0.30$, reflecting its core role in the system’s effectiveness. The Industrial Firewall and the Station Switch, being critical infrastructure for maintaining system security and connectivity, are assigned the next highest weights, set at $w_{1}=0.20$ and $w_{4}=0.16$, respectively. The IDS and the Communication Gateway, providing crucial support for threat identification and information transmission, are assigned weights of $w_{2}=0.12$ and $w_{5}=0.12$. The two protection devices are assigned relatively lower weights, both set to $w_{6}=w_{7}=0.05$, primarily reflecting their specialized and localized characteristics in protecting specific equipment and isolating faults.

To characterize the contribution of a single device in different health states to the overall system effectiveness, we define the single-device effectiveness function $\varphi_{i}\left(X_{i}\right)$ as the mapping relationship between the device state and its functional availability. Considering that device performance in ICS typically degrades monotonically with deteriorating health status, while possessing certain redundancy and fault tolerance, the mapping function is defined as follows:

$$\varphi_{i}\left(X_{i}\right)=\left\{\begin{array}{cc} 1.0, & X_{i}=0\;(\mathrm{Normal}\;\mathrm{Operation})\\ 0.8, & X_{i}=1\;(\mathrm{Minor}\;\mathrm{Anomaly})\\ 0.3, & X_{i}=2\;(\mathrm{Severe}\;\mathrm{Anomaly})\\ 0, & X_{i}=3\;(\mathrm{Complete}\;\mathrm{Failure}) \end{array}\right.$$

This definition reflects the impact pattern of varying degrees of failure on functional availability: when a device is only slightly anomalous, its main functions can still be maintained, albeit with slightly degraded performance; when it enters a severely anomalous state, functional attenuation becomes significant, providing only limited support, and in a failed state, its contribution is entirely lost.

It is important to note that the range of the system effectiveness function is [0,1], representing the comprehensive operational capability of the system at the current moment. When all devices are normal, F=1, indicating the system is in the optimal operational state. When some key devices are anomalous, the extent of the effectiveness value decrease depends on their weights and state severity. Therefore, this function not only reflects the overall performance level of the system but also provides a measurement benchmark for quantifying the impact of attack–defense actions and comparing different defense strategies. Furthermore, although the contribution of each device to the overall system’s effectiveness is calculated separately in the system effectiveness function, the interdependencies between devices are still reflected in the distribution of device states through the DBN described in Section 3.2.1 thereby influencing each device’s contribution to the overall effectiveness through the state distribution.

### 3.4. Experiments and Analysis

To demonstrate the capability of the proposed dynamic evaluation framework in pre-engagement planning scenarios, two sets of experiments are designed and executed based on the substation automation system described in Section 3.1. All experiments are conducted using the effectiveness computation framework from Section 2, performing probabilistic inference on the DBN model.

#### 3.4.1. Defense Strategy Evaluation and Ablation Study

The core objective of this experiment is to verify whether the evaluation framework can quantify the potential effects and uncertainties of different defense strategies under the defender’s information incompleteness, thereby providing a scientific basis for pre-engagement strategy selection. The experiment systematically analyzes the effects of defense strategies by simulating the defender’s decision-making process under different observed states and threat landscapes.

(1) Experimental Settings

The experiment is designed around three core variables: the defender’s observed state, available defense strategies, and the assumed attack conditions.

**(a) Observed States.** To reflect the defender’s decision-making characteristics under different system health conditions, three typical observed states are set:

- Z(t)=(0,0,0,0,0,0,0), denoted $Z^{0}$, indicating the defender observes the system in an entirely normal state.
- Z(t)=(1,0,0,0,0,0,0), denoted $Z^{1}$, indicating the defender observes a minor anomaly in the firewall.
- Z(t)=(0,0,2,0,0,0,0), denoted $Z^{2}$, indicating the defender observes a severe anomaly in the SCADA Server.

These scenarios are selected to span the operational spectrum from normal conditions through a boundary device anomaly to a severe core device degradation, representing the range of system conditions under which pre-engagement defense evaluation is typically required.

**(b) Defense Strategy Combinations.** To conduct a comparative analysis of defense strategy effects, the following eight defense strategy combinations are selected:

- The defender takes no action, i.e., D(t)=(0,0,0,0,0). This strategy set is denoted $D^{0}$, serving as the baseline.
- The defender individually enables defense strategies targeting the firewall, IDS, SCADA Server, switch, and Communication Gateway, resulting in five strategy sets. These are denoted $D^{i}$ (i=1,2,…,5), where for strategy $D^{i}$, the corresponding D(t) has $D_{i}\left(t\right)=1,D_{j}\left(t\right)=0$ (1≤i,j≤5,j≠i).
- The defender simultaneously protects the firewall and SCADA Server, i.e., D(t)=(1,0,1,0,0), denoted $D^{1,3}$.
- The defender simultaneously protects the IDS and Communication Gateway, i.e., D(t)=(0,1,0,0,1), denoted $D^{2,5}$.

These eight combinations span the logical progression from no-defense through individual strategies to combined strategies, and are representative of the types of defense configurations a defender would consider in pre-engagement planning.

**(c) Attack Conditions.** To systematically evaluate the impact of attacker strategy uncertainty, three attack conditions are defined:

- Unknown Attack ($A^{x}$): The defender has no knowledge of which attack strategies will be employed. The effectiveness distribution is computed by marginalizing over all possible attack strategy combinations according to P(A∣X). This condition retains full attacker strategy uncertainty.
- No Attack ($A^{0}$): The attacker is known to employ no attack strategies (A=(0,0,0,0,0)). This eliminates attacker strategy uncertainty entirely.
- Full-Scale Attack ($A^{1}$): The attacker is known to employ all five attack strategies simultaneously (A=(1,1,1,1,1)). This also eliminates attacker strategy uncertainty, but under a worst-case assumption.

The comparison among $A^{0}$, $A^{x}$, and $A^{1}$ thus isolates the contribution of attacker strategy uncertainty to total prediction uncertainty.

For scenarios $A^{0}$ and $A^{1}$, the attacker strategy is treated as known evidence, and the effectiveness distribution is computed as P(ΔF=δ∣Z(t),D(t),A(t)) with A(t) fixed to the specified values. For $A^{x}$, the inference engine marginalizes over the distribution P(A∣X).

**(d) Model Variants for Ablation Study.** To provide a principled baseline comparison and quantify the contribution of the remaining modeling components, two reduced variants of the full model are constructed:

- $M_{\text{no-obs}}$: The observation model is replaced by an identity mapping ($P(Z_{i}=k|X_{i}=k)$=1), eliminating partial observability. This variant is functionally equivalent to a standard DBN evaluation that assumes the defender has full knowledge of the true system state—the common assumption in existing DBN-based risk assessment methods.
- $M_{\text{no-dep}}$: Inter-device dependency propagation is removed by making the state of each device conditionally independent of others. This variant represents the device-independent modeling adopted by most existing frameworks, which do not capture ICS-specific functional dependencies.

Both variants are evaluated under the unknown attack condition $A^{x}$ across the same observation scenarios and defense strategies defined above. Together with the full model, they form a controlled ablation setup in which each component—observation uncertainty, dependency propagation, and attacker strategy uncertainty—can be isolated and its individual contribution measured.

(2) Strategy Evaluation with the Full Framework

This subsection evaluates the proposed framework’s ability to discriminate among defense strategies and to reveal structural dependencies. The analysis proceeds under the unknown attack condition $A^{x}$ across three observation scenarios, first comparing individual and combined strategies by expectation and entropy, then examining synergistic effects, and finally demonstrating the complementarity of the two metrics.

**Figure 7:**
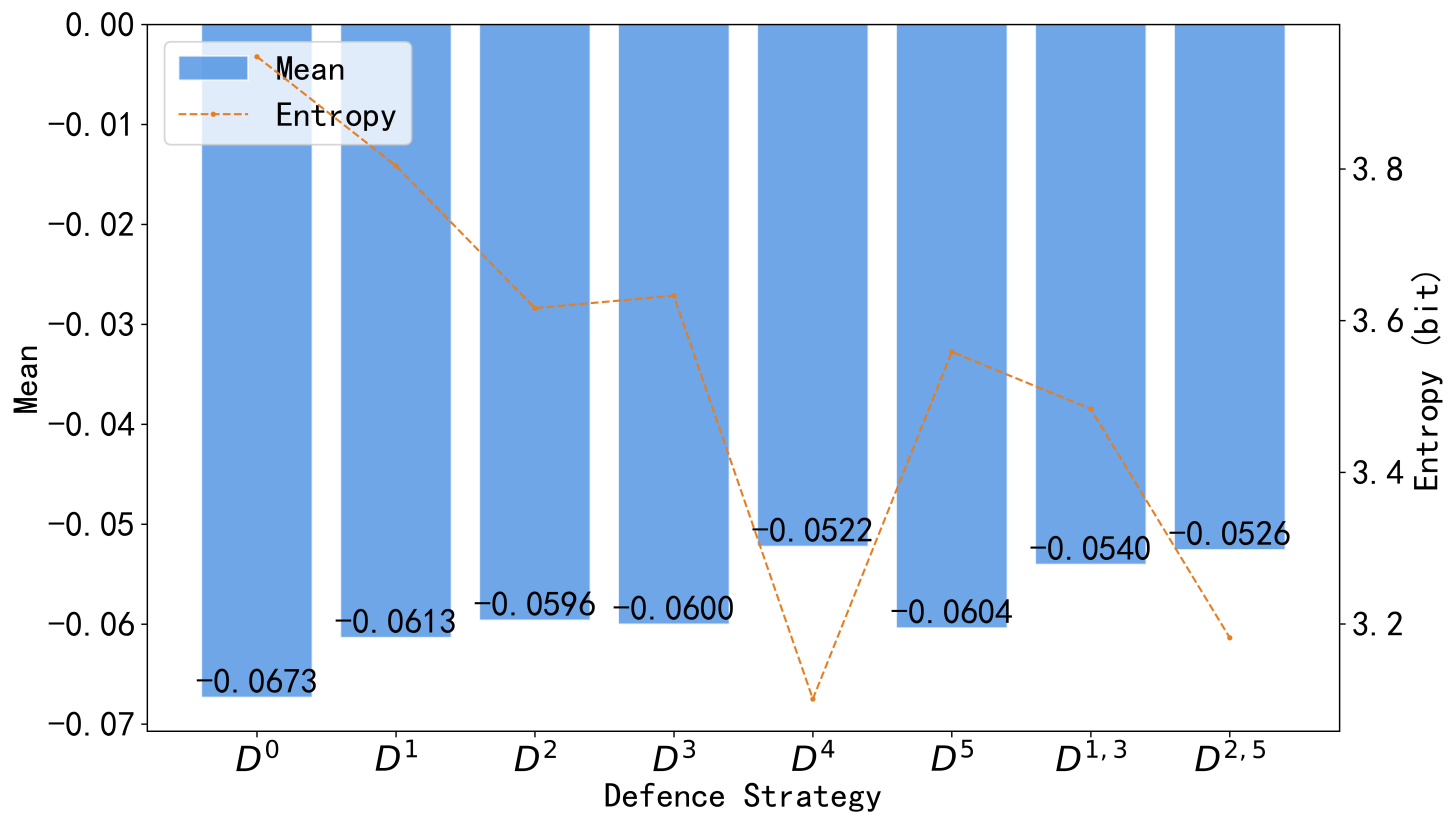
Expectation and entropy of effectiveness differential for different defense strategies under the $Z^{0},A^{x}$ scenario.

**(a) Comparison of Defense Strategies Under Unknown Attack.** To verify the framework’s ability to discriminate among strategies, the expectation E(ΔF) and information entropy H(ΔF) are computed for all eight defense combinations under the unknown attack condition $A^{x}$. Figure 7 shows the results for the all-normal observation $Z^{0}$. The framework clearly distinguishes among strategies: the baseline $D^{0}$ yields the lowest expected effectiveness (E=−0.0673), while the combined strategies $D^{1,3}$ and $D^{2,5}$ produce the highest (E=−0.0540 and −0.0526, respectively).

Notably, the best-performing single-device strategy is $D^{4}$ (E=−0.0522), which protects the Station Switch—a device with a moderate weight of 0.16. This strategy outperforms the protection of higher-weight devices such as the SCADA Server ($D^{3}$, E=−0.0600, weight 0.30) and the firewall ($D^{1}$, E=−0.0613, weight 0.20). This counterintuitive result illustrates that a device’s structural position in the dependency network can amplify the benefit of its protection beyond what its weight alone would suggest: the Station Switch serves as a communication hub for multiple downstream devices, and keeping it operational suppresses cascading failure risks across a broad portion of the system. The following subsections further explore how such structural effects shift strategy rankings under anomalous observations and give rise to defense synergies.

**Figure 8:**
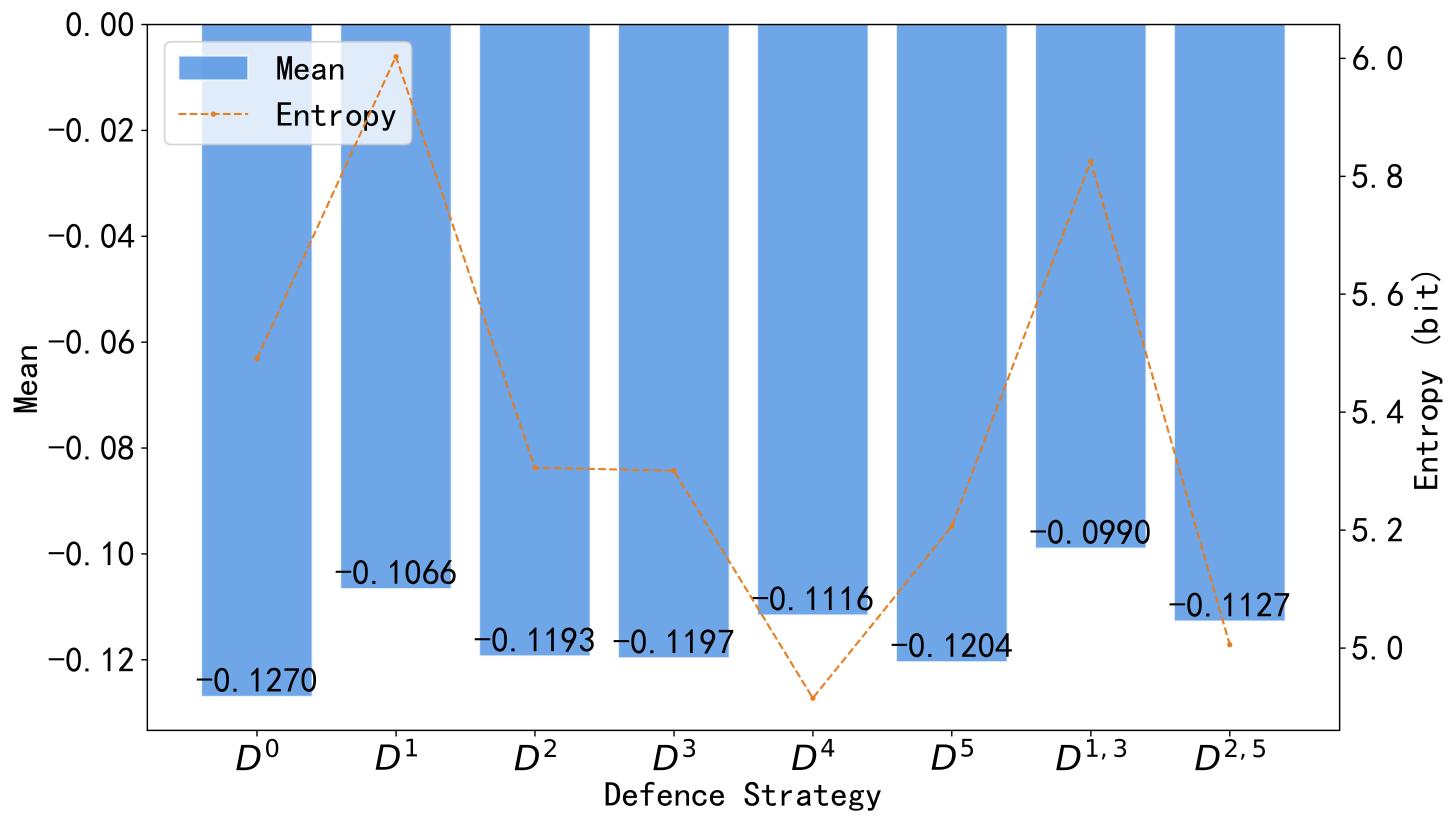
Expectation and entropy of effectiveness differential for different defense strategies under the $Z^{1},A^{x}$ scenario.

**Figure 9:**
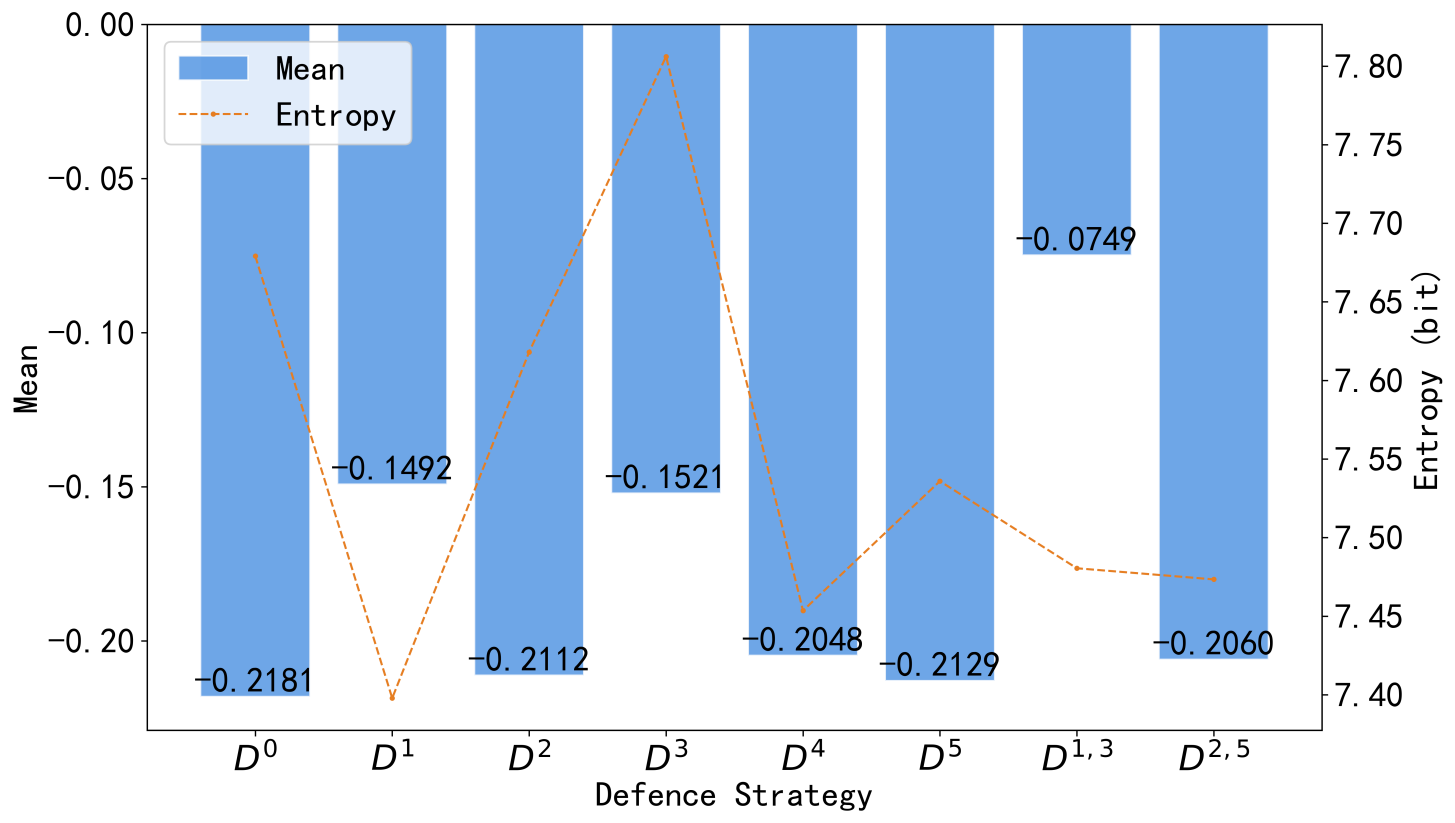
Expectation and entropy of effectiveness differential for different defense strategies under the $Z^{2},A^{x}$ scenario.

**(b) Effectiveness Analysis in Anomaly Scenarios**Figure 8 and Figure 9 present the evaluation results under the firewall minor anomaly ($Z^{1}$) and SCADA Server severe anomaly ($Z^{2}$) scenarios, both under the unknown attack condition $A^{x}$. The results reveal that the optimal defense strategy depends heavily on the current system state, highlighting the value of dynamic evaluation.

Under $Z^{1}$, the strategy $D^{1}$ (directly targeting the anomalous firewall) yields the highest expected effectiveness among all single-device strategies, which aligns with the intuitive principle of “addressing the anomaly where it occurs.”

Under $Z^{2}$, however, a more insightful outcome emerges: the firewall protection strategy $D^{1}$ outperforms the direct SCADA restoration strategy $D^{3}$. This counterintuitive ranking—which emerges when the system is under stress but not when all devices are normal—illustrates that in a tightly coupled ICS, the global benefit of securing a network boundary chokepoint can exceed the local benefit of repairing the most severely damaged device. The firewall, by blocking further threat propagation, creates a more favorable environment for system recovery, whereas directly restoring the SCADA Server without perimeter hardening leaves the repaired system exposed to continued attacks. This finding underscores the importance of system-wide dependency analysis, which the proposed DBN framework is designed to provide.

Beyond strategy rankings, comparing the two anomaly scenarios reveals that the potential system gain from restoring a severely anomalous device (e.g., SCADA in $Z^{2}$) is much larger than that from restoring a mildly anomalous one (e.g., firewall in $Z^{1}$), owing to the larger state value transition (from 0.3 to 1.0) and the higher device weight ($w_{3}=0.30$).

**Figure 10:**
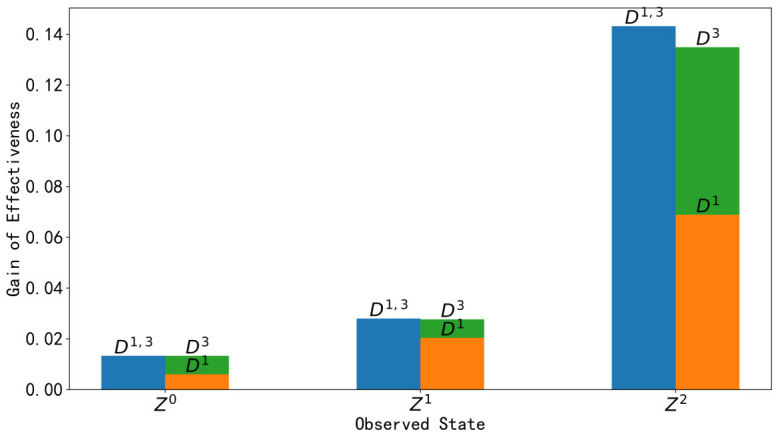
Defense effectiveness gain of the combination of $D^{1}$ and $D^{3}$.

**Figure 11:**
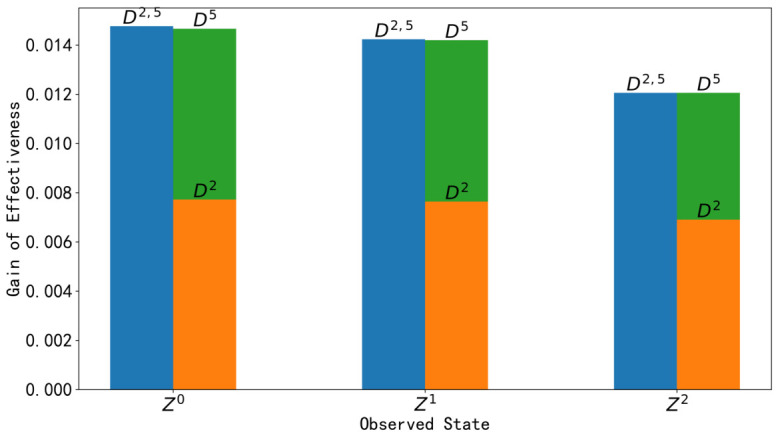
Defense effectiveness gain of the combination of $D^{2}$ and $D^{5}$.

**(c) Effectiveness Gain of Combined Defense Strategies.** To quantify the synergistic effect of combined strategies, the effectiveness gain of a strategy *D* relative to the no-defense baseline $D^{0}$ is defined as $G=\Delta F_{D}-\Delta F_{0}$. For two single strategies $D^{i}$ and $D^{j}$, synergy is identified when the combined gain satisfies $G_{i,j}>G_{i}+G_{j}$. Figure 10 and Figure 11 report the gains for the two strategy groups $\left(D^{1},D^{3},D^{1,3}\right)$ and $\left(D^{2},D^{5},D^{2,5}\right)$.

Under $Z^{0}$ and $Z^{1}$, the combined gains are approximately additive ($G_{1,3}\approx G_{1}+G_{3}$, $G_{2,5}\approx G_{2}+G_{5}$), indicating that no measurable synergy emerges in these two scenarios under the current parameterization.

Under $Z^{2}$, a modest but clear synergistic effect emerges for the firewall–SCADA combination. The combined gain $G_{1,3}=+0.1432$ exceeds the sum of individual gains $G_{1}+G_{3}=+0.1349$ by approximately 6%. This synergy arises because the two strategies complement each other through the ICS dependency network, as discussed above. The pair ($D^{2},D^{5}$) does not exhibit synergy in any of the three observation scenarios under the current parameterization.

This dynamic capability—quantifying single-strategy effects and identifying state-dependent synergistic potential grounded in system structure—provides actionable guidance for moving beyond static, device-centric defense planning toward system-aware strategy selection.

**Figure 12:**
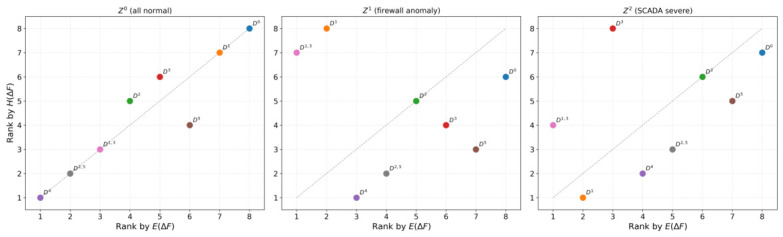
Rankings by E(ΔF) and by H(ΔF).

**(d) Entropy and Expectation as Complementary Metrics.** To further assess the extent to which entropy and expectation differ in their assessment of strategies, Figure 12 compares the rankings of all eight strategies by E(ΔF) and by H(ΔF) under the unknown attack condition $A^{x}$. If the two metrics were redundant, all points would lie on the diagonal.

Under $Z^{0}$, the rankings by expectation and by entropy are nearly identical—when the system appears healthy, the strategies that perform best on average also tend to produce the most predictable outcomes. Under $Z^{1}$ and $Z^{2}$, however, the two rankings diverge substantially. In $Z^{1}$, strategies $D^{1,3}$ and $D^{1}$ rank first and second by expectation but fall to seventh and eighth by entropy; in $Z^{2}$, $D^{3}$ ranks third by expectation but last by entropy. These divergences do not indicate that one ranking is correct and the other is not. Rather, they reveal a structural trade-off: strategies that target the fault source can achieve high expected gains, yet the stochastic nature of attack success and dependency propagation makes their outcomes less predictable. A risk-averse defender may therefore prefer a strategy with slightly lower expected effectiveness but substantially higher confidence in its outcome.

This trade-off is most pronounced when the system is under stress—precisely the condition under which pre-engagement defense evaluation is most needed. The proposed framework reports both E(ΔF) and H(ΔF) without prescribing how they should be weighted, enabling the defender to balance expected performance against prediction confidence according to their risk preference.

These findings are obtained under the specific CPT configuration and network topology of the substation case study. While the qualitative insights are expected to hold across similar ICS topologies, the specific numerical rankings are conditioned on the chosen parameters and should be interpreted as illustrative rather than prescriptive.

(3) Ablation Study: Contribution of Uncertainty Sources

This section systematically isolates the contribution of each modeling component. The analysis proceeds from the outermost source of uncertainty to the innermost:

- Attacker strategy uncertainty is examined through the $A^{0}$–$A^{x}$–$A^{1}$ contrast, which varies the defender’s knowledge of which attacks will be launched while keeping the full DBN model unchanged.
- Observation uncertainty is isolated by comparing the full model against $M_{\text{no-obs}}$ under $A^{x}$, where the observation noise is replaced by perfect state knowledge.
- Dependency propagation is assessed by comparing the full model against $M_{\text{no-dep}}$ under $A^{x}$, where inter-device dependencies are removed.
- Entropy vs. variance is examined in light of the preceding comparisons, to evaluate whether the entropy metric provides information distinct from traditional variance-based measures.

State evolution uncertainty—the inherent randomness in whether an attack succeeds or a device degrades—is the foundational aleatoric component of the DBN and is common to all probabilistic approaches. It is therefore not separately ablated.

**Figure 13:**
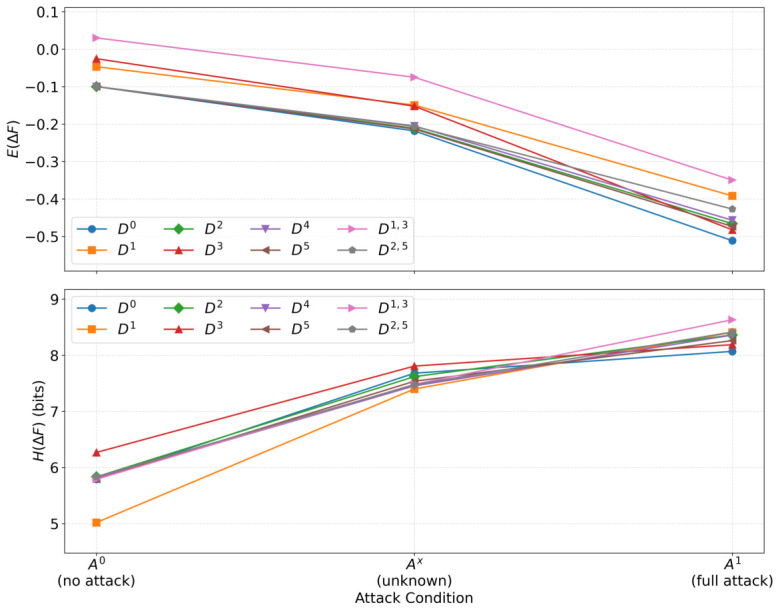
E(ΔF) and H(ΔF) across attack conditions under $Z^{2}$.

**(a) Attack Strategy Uncertainty ($A^{0}$ vs. $A^{x}$ vs. $A^{1}$).** The three attack conditions differ in how they treat attacker strategy: $A^{0}$ and $A^{1}$ fix the attacker’s actions to known states, while $A^{x}$ retains the full uncertainty by marginalizing over P(A∣X). Figure 13 shows the resulting E(ΔF) and H(ΔF) for all eight defense strategies under $Z^{2}$; the same monotonic trends hold under $Z^{0}$ and $Z^{1}$.

The steady decline in E(ΔF) and the corresponding increase in H(ΔF) from $A^{0}$ through $A^{x}$ to $A^{1}$ quantify the contribution of attacker strategy to total prediction uncertainty. Notably, $A^{1}$ yields the highest entropy (8.07 bits for $D^{0}$), exceeding that of $A^{x}$ (7.68 bits). This counterintuitive ordering reveals a nuanced interplay: although both $A^{0}$ and $A^{1}$ eliminate attacker strategy uncertainty, they differ fundamentally in the aleatoric perturbation they introduce. $A^{1}$ activates all five attacks simultaneously, each with its own success probability, thereby maximizing the aleatoric perturbation. The unknown attack condition $A^{x}$, by marginalizing over all possible attack configurations, computes a probability-weighted average that blends heterogeneous risk pathways into an intermediate entropy. This highlights a key implication: models that assume a fixed worst case ($A^{1}$) may overstate the likely prediction uncertainty, while those assuming no attack ($A^{0}$) systematically underestimate it. The proposed framework, by accommodating the full spectrum within a single inference procedure, allows the defender to assess strategy effectiveness under realistic threat ambiguity.

As a supplementary observation, the entropy under $A^{0}$ is not zero when the system is observed to be anomalous (H=5.79 bits for $D^{0}$ under $Z^{2}$), whereas it drops to nearly zero under the all-normal observation (H=0.01 bits for $D^{0}$ under $Z^{0}$). This contrast confirms that the residual uncertainty under $A^{0}$ arises not from attack activity, but from the anomalous observation itself: $Z^{2}$ strongly suggests a degraded current state, and inter-device dependencies can propagate this existing fault even without any external attack. This finding underscores the necessity of modeling dependency propagation independently of attacker behavior.

**Figure 14:**
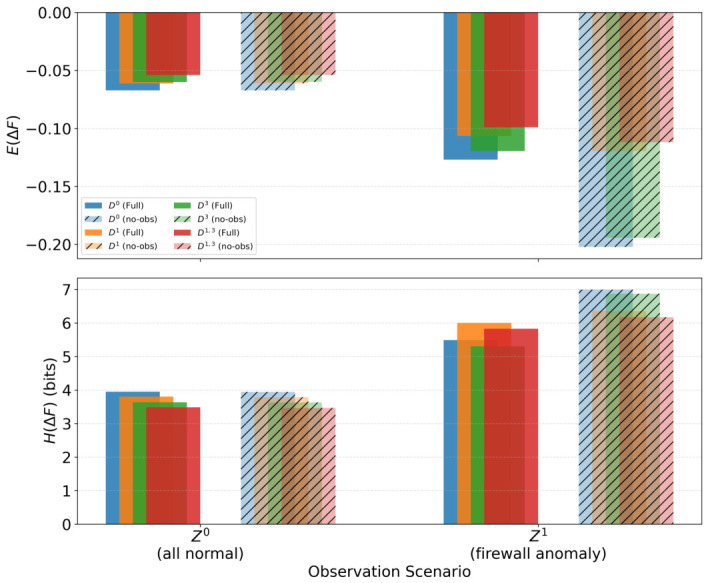
E(ΔF) and H(ΔF) for $M_{\mathrm{full}}$ and $M_{\text{no-obs}}$ under $Z^{0}$ and $Z^{1}$.

**(b) Observation Uncertainty ($M_{\mathrm{full}}$ vs. $M_{\text{no-obs}}$).** To isolate the effect of partial observability, the full model is compared against $M_{\text{no-obs}}$, in which the observation model is replaced by an identity mapping. Figure 14 presents the results for four representative strategies under $Z^{0}$ and $Z^{1}$. Strategies $D^{2}$, $D^{4}$, $D^{5}$, and $D^{2,5}$ behave similarly to $D^{0}$ and $D^{3}$, forming part of the large-difference group discussed below.

Under $Z^{0}$ (all normal), the two models produce nearly identical expectations and entropies across all strategies. This is because the low observation error rate and the already-normal readings concentrate the posterior around the true system state even without perfect observation. Removing observation uncertainty therefore adds negligible information when the system appears healthy.

Under $Z^{1}$ (firewall minor anomaly), removing observation uncertainty produces a substantial but uneven effect that operates at two levels:

- **Global elevation of prediction uncertainty.** The expected loss and entropy increase across all strategies. In the full model, the posterior correctly reflects the defender’s cognitive limitations: the observation noise mixes a dominant fault branch ($X_{1}=1$) with a low-probability false-alarm branch ($X_{1}=0$), the latter carrying substantially lower downstream uncertainty because a healthy firewall implies weaker attack incentives and reduced dependency propagation. Removing observation uncertainty ($M_{\text{no-obs}}$) collapses the posterior onto the fault branch, assigning full weight to the high-uncertainty configuration. This systematically elevates the predicted uncertainty and expected cost for every strategy. For the baseline $D^{0}$, the expected loss drops from −0.1270 to −0.2022 and the entropy rises from 5.49 to 6.99 bits; the same pattern holds for $D^{2}$–$D^{5}$ and $D^{2,5}$.
- **Uneven impact across strategies.** This elevation is not uniform. Strategies that directly protect the anomalous device ($D^{1}$ and $D^{1,3}$) largely restore $X_{1}$ to normal, thereby switching the downstream uncertainty back to the low-uncertainty regime and insulating themselves from the global shift (ΔE≈0.013, ΔH≈0.35 bits). Strategies that do not target the anomaly ($D^{0},D^{2}$–$D^{5},D^{2,5}$) absorb the full impact, widening the apparent performance gap between relevant and irrelevant options. The result is a distortion of the relative strategy ranking that is absent in the full model, where the false-alarm branch narrows these differences.

This two-level effect highlights the value of improved situational awareness for pre-engagement defense planning. Under perfect observation, the performance gap between strategies that target the true fault and those that do not becomes clearly visible, enabling the defender to discriminate among options that would appear similarly effective under noisy observations. The inflated prediction uncertainty under perfect observation is not a drawback—it is an accurate reflection of the full aleatoric uncertainty that noisy observations partially mask. However, when the defender’s actual monitoring capabilities are imperfect, a model that assumes perfect observation would evaluate strategies under an information state that the defender does not occupy, leading to assessments that are internally consistent but externally misaligned with the defender’s genuine decision context. The full model, by faithfully retaining the observational limitations that exist in practice, yields strategy assessments that are unbiased with respect to the defender’s actual information constraints. These results demonstrate that explicitly modeling observation noise is essential not as an end in itself, but as a means of grounding pre-engagement evaluations in the reality of the defender’s situational awareness.

**Figure 15:**
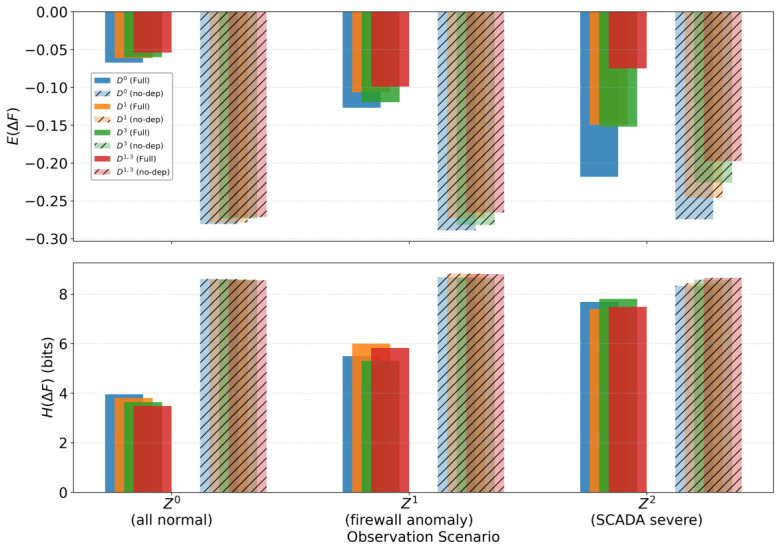
E(ΔF) and H(ΔF) for the full model and $M_{\text{no-dep}}$ under $Z^{0}$, $Z^{1}$, and $Z^{2}$.

**(c) Dependency Propagation ($M_{\mathrm{full}}$ vs. $M_{\text{no-dep}}$).** To isolate the role of inter-device dependencies, the full model is compared against $M_{\text{no-dep}}$, in which all device-to-device dependency links are removed while retaining the attack strategy uncertainty under $A^{x}$. Figure 15 presents the results for four representative strategies ($D^{0}$, $D^{1}$, $D^{3}$, $D^{1,3}$) across the three observation scenarios.

**Dependencies as probabilistic constraints on the state space.** The surge in entropy upon removing dependencies directly demonstrates that inter-device dependencies act as powerful constraints on prediction uncertainty. In the full model, the dependency structure couples the states of connected devices, restricting the system to a limited set of internally consistent joint states and keeping the entropy relatively low. When these constraints are lifted in $M_{\text{no-dep}}$, all devices evolve independently, and the set of possible joint states expands combinatorially, driving the entropy toward its theoretical maximum. The effect is visible across all observation scenarios and all strategies.

Notably, the magnitude of this constraint is conditional on the system state. In the full model, the entropy of $D^{0}$ already rises from 3.95 ($Z^{0}$) to 5.49 ($Z^{1}$) to 7.68 ($Z^{2}$), because each successive anomaly activates additional propagation pathways through the dependency network. The constraints that tightly restrict the state space under healthy conditions are progressively released as faults accumulate. Consequently, the marginal effect of removing dependencies diminishes from $Z^{0}$ (ΔH=+4.66) through $Z^{1}$ (ΔH=+3.19) to $Z^{2}$ (ΔH=+0.65), following the same monotonic trend across all strategies.

**Dependencies as the physical basis for defense synergy.** Under $Z^{2}$, the synergistic effect of the combined strategy $D^{1,3}$ identified in the full model (Section Strategy Evaluation with the Full Framework) vanishes entirely when dependencies are removed. Table 2 reports the gains of $D^{1}$, $D^{3}$, and $D^{1,3}$ relative to $D^{0}$ under both models. In $M_{\text{no-dep}}$, the combined gain (+0.0769) falls precisely to the sum of the individual gains (+0.0283+0.0486), demonstrating strict additivity. This confirms that the inter-device dependency graph is the physical mechanism through which defense strategies can reinforce each other. Without these functional links, each strategy operates on its target device in isolation, and their joint effect is merely the sum of their independent contributions.

**Dependencies as a redistributor of strategy effectiveness.** The same table reveals a deeper structural effect. In the full model, the gains of $D^{1}$ and $D^{3}$ are nearly equal (+0.0689 vs. +0.0660). In $M_{\text{no-dep}}$, the gain of $D^{1}$ drops sharply to +0.0283, while that of $D^{3}$ declines more moderately to +0.0486. This divergence arises because a substantial portion of $D^{1}$’s value in the full model comes from its structural position: protecting the firewall blocks external threats from propagating through the dependency network to downstream devices. When dependencies are severed, this indirect protective effect is lost, and $D^{1}$ retains only the direct benefit of safeguarding a device with a moderate weight ($w_{1}=0.20$). In contrast, $D^{3}$ directly protects the SCADA Server—the device with the highest weight ($w_{3}=0.30$)—so its direct value remains substantial even without dependency-mediated amplification. Dependency propagation thus does not merely transmit risk; it actively redistributes the effectiveness of defense strategies, and ignoring it would systematically misjudge the relative value of protecting different system components.

Taken together, these three findings establish the dual role of inter-device dependencies in ICS defense evaluation. Dependencies function simultaneously as conduits for cascading failures, as constraints that make prediction uncertainty tractable, and as channels through which defense strategies achieve structural amplification. A model that omits dependency propagation would overstate prediction uncertainty, miss synergistic defense opportunities, and misrank the effectiveness of individual strategies.

**Figure 16:**
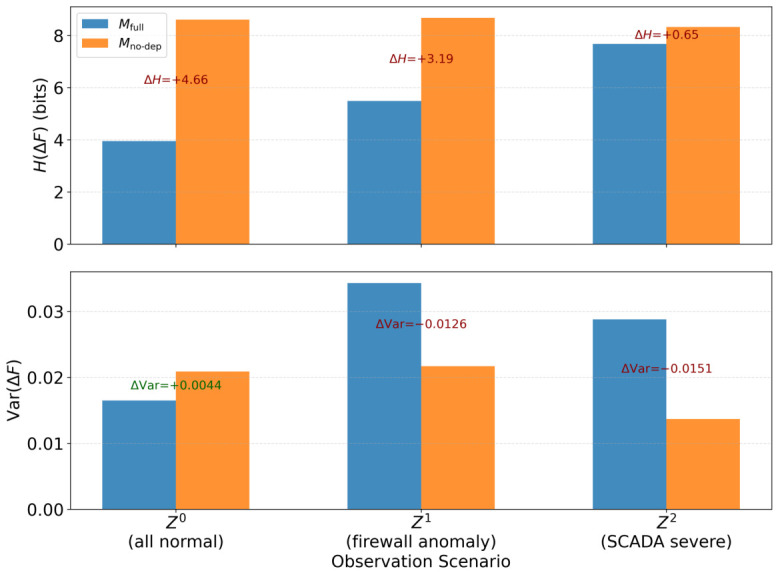
H(ΔF) and Var(ΔF) for $D^{0}$ under the full model and $M_{\text{no-dep}}$.

**(d) Entropy vs. Variance.** In the attack strategy and observation uncertainty experiments, the entropy and variance of ΔF consistently move in the same direction. The dependency propagation experiment, however, reveals a systematic divergence. Under $Z^{0}$, both metrics increase when dependencies are removed, but their magnitudes differ starkly: entropy surges from 3.95 to 8.61 bits, whereas variance increases only marginally from 0.0165 to 0.0209. Under $Z^{1}$ and $Z^{2}$, the divergence becomes directional: entropy increases (from 5.49 to 8.68 and from 7.68 to 8.33 bits) while variance decreases (from 0.0343 to 0.0217 and from 0.0288 to 0.0137). Figure 16 visualizes this opposite-direction movement for the baseline strategy $D^{0}$ across the three scenarios. This pattern holds across all eight strategies, confirming that it is a systematic consequence of removing dependency constraints.

This divergence arises because the two metrics capture fundamentally different properties of the effectiveness distribution. Variance is dominated by the numerical scale of extreme outcomes: cascading failures in the full model produce heavy-tailed losses that inflate variance, whereas independent devices in $M_{\text{no-dep}}$ produce more uniformly distributed, less extreme losses. Entropy, by contrast, reflects the overall shape and dispersion of the probability distribution irrespective of the numerical encoding of outcomes. In ICS contexts where effectiveness values are elicited from domain experts and may be revised, an encoding-independent metric offers greater stability. The proposed framework can compute and report both measures; entropy is adopted as the default companion metric for its direct information-theoretic interpretation of prediction uncertainty and its sensitivity to the structural effects identified above.

#### 3.4.2. CPT Noise Robustness Analysis

In practical ICS, obtaining all CPTs in the DBN with perfect accuracy is highly challenging and often subject to certain errors. To examine the robustness and reliability of the proposed framework under imprecise parameters, this experiment systematically analyzes the stability of evaluation results when core CPTs are perturbed.

(1) Experimental Settings

Four types of CPTs ($I_{1}$ to $I_{4}$) are perturbed independently with relative amplitudes in [0,2.5%], [2.5%,5%], [5%,10%], and [10%,15%]. For each CPT, the perturbation procedure is as follows:

1. The entry with the largest probability value $p_{\max}$ is selected as the anchor;
2. A target relative change ϵ is drawn uniformly from the specified interval;
3. The anchor entry is adjusted to $p_{\max}^{\prime }=p_{\max}\cdot \left(1\pm \epsilon \right)$, with the sign chosen randomly;
4. The difference $\Delta =p_{\max}-p_{\max}^{\prime }$ is redistributed among the remaining entries in proportion to their original values, ensuring that the sum of all probabilities remains exactly 1.0 and that no entry falls outside [0,1].

The reported perturbation amplitude for each run is computed as $\epsilon =|p_{\max}-p_{\max}^{\prime }|/p_{\max}$. This procedure perturbs the entire distribution simultaneously while using the largest probability entry merely as a convenient scalar reference for quantifying perturbation intensity.

The expectation and entropy of the effectiveness differential ($E_{0}\left(\Delta F\right)$ and $H_{0}\left(\Delta F\right)$) computed using the original, unperturbed CPTs (denoted $P_{0}$) under different scenarios (e.g., combinations of observed states and defense strategies) serve as the baseline. For each set of conditional probabilities from $I_{1}$ to $I_{4}$ and each noise interval, 30 independent Monte Carlo simulations are performed. In each simulation, the evaluation results E(ΔF) and H(ΔF) after perturbation are computed, and their relative deviations from the baseline are calculated:

$$\mathrm{Relative}\;\mathrm{Bias}\;\mathrm{of}\;\mathrm{expectation}=\frac{{|E(\Delta F)-E}_{0}\left(\Delta F\right)|}{{|E}_{0}\left(\Delta F\right)|}$$

$$\mathrm{Relative}\;\mathrm{Bias}\;\mathrm{of}\;\mathrm{Entropy}=\frac{{|H(\Delta F)-H}_{0}\left(\Delta F\right)|}{|H_{0}\left(\Delta F\right)|}$$

Finally, the deviation values for each condition are averaged over the 30 simulations.

(2) Results and Analysis

**Figure 17:**
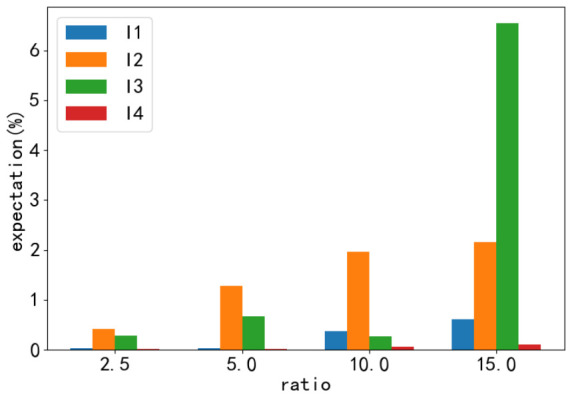
Relative bias of expectation under CPT noise.

**Figure 18:**
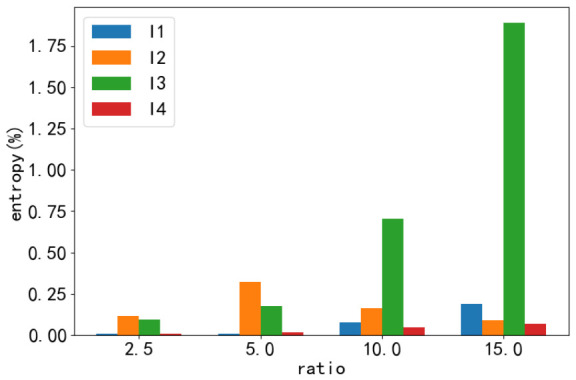
Relative bias of entropy under CPT noise.

Figure 17 and Figure 18 show the relative biases of the expectation and entropy of the effectiveness differential, respectively, as a function of CPT noise level under typical observation scenarios. Analysis of the charts leads to the following conclusions:

**Overall Robustness.** Under noise levels up to 15%, the relative bias of the expectation E(ΔF) and the entropy H(ΔF) across all tested parameter types ($I_{1}$–$I_{4}$) remains within 7% and 2%, respectively. This indicates that the evaluation framework possesses good robustness against errors in CPT parameters, with essentially stable evaluation results.

**Sensitivity of Uncertainty Metric.** Compared to E(ΔF), H(ΔF) demonstrates lower sensitivity to parameter perturbations in most cases. The perturbation procedure randomly applies either a positive or a negative adjustment to the largest probability entry in each CPT: a positive perturbation increases the concentration of the distribution, reducing entropy, while a negative perturbation redistributes probability mass more evenly, increasing entropy. Since the perturbation direction is chosen at random across runs, these two effects partially cancel each other when the relative biases are averaged.

**Parameter Sensitivity Differences.** Since the relative biases of the entropy H(ΔF) are consistently small across all parameter types (remaining below 2% even at 15% perturbation, as discussed above), the differences among $I_{1}$–$I_{4}$ are more clearly observed through the expectation E(ΔF). The following analysis therefore focuses on the expectation results.

The perturbation experiment reveals that the relative importance of the four parameter types depends markedly on the perturbation level. Two main patterns emerge from the data. First, at low-to-moderate perturbation amplitudes, the observation likelihood $I_{2}$ consistently exerts the strongest influence on the expectation E(ΔF), whereas at high perturbation amplitudes, the attacker strategy model $I_{3}$ becomes the dominant error source: at 15% perturbation, the expectation relative bias of $I_{3}$ reaches 6.55%, far exceeding that of $I_{2}$ (2.15%). Second, the prior distribution $I_{1}$ and the state transition probability $I_{4}$ consistently exhibit the weakest impact across all tested perturbation levels, with both remaining below 1% even at 15% perturbation.

These different sensitivities can be traced to the computational roles of the parameters. $I_{2}=P\left(Z\left(t\right)|X\left(t\right)\right)$ sits at the very beginning of the inference chain, defining how observations map to state beliefs in Step 1. Its influence on the evaluation outcome grows approximately linearly with perturbation amplitude, reflecting its position in a single-step Bayesian update. $I_{3}=P\left(A\left(t\right)|X\left(t\right)\right)$, by contrast, determines the probability weights assigned to each possible attack strategy combination in the marginalization of Equation (3). Because this marginalization sums over the entire attack strategy space, disrupting these weights redistributes probability mass across qualitatively different attack scenarios, producing a nonlinear amplification of error that becomes pronounced at higher perturbation levels. This nonlinear growth causes $I_{3}$ to surpass $I_{2}$ as the dominant error source when perturbations are large.

The consistently low sensitivity of $I_{1}$ and $I_{4}$ stems from different structural mechanisms. $I_{1}$ appears in two places in the inference chain: first in Step 1, where its influence is tempered by the observation likelihood $I_{2}$ through Bayes’ rule; second, in Step 2, where it occupies a downstream position alongside $I_{4}$ in the state transition CPTs. $I_{4}$ is further constrained by its terminal position in the inference chain and by physical laws that restrict the direction of its probability fluctuations (e.g., “state cannot improve when only an attack exists”). The combination of these factors keeps both $I_{1}$ and $I_{4}$ stably low in their impact.

Notably, the low sensitivity of $I_{1}$ should not be interpreted as evidence that inter-device dependencies are unimportant. The prior distribution $I_{1}$ encodes the dependency structure among devices through its factorization into conditional probability terms. The CPT perturbation analysis demonstrates that the framework is robust to moderate inaccuracies in the numerical values of these dependency CPTs, while the ablation study (Section Ablation Study: Contribution of Uncertainty Sources) shows that removing the dependency structure entirely causes entropy to surge and strategy rankings to be fundamentally reshaped. Together, these results indicate that the structural presence of dependencies matters far more than their precise parameterization. More broadly, the perturbation experiments and the ablation study provide complementary perspectives on the framework’s robustness—parameter-level and structure-level perspectives, respectively.

These findings have direct implications for modeling priorities. The observation likelihood ($I_{2}$) and the attacker strategy model ($I_{3}$) are the two parameters that most critically govern evaluation reliability. This aligns with the foundational premises of the proposed framework: that accurate observability modeling—as opposed to assuming a global, omniscient perspective—is essential for pre-engagement evaluation under incomplete information, and that careful characterization of adversary behavior is indispensable for robust strategy assessment.

### 3.5. Computational Performance

The computational efficiency of the proposed two-stage strategy is evaluated with respect to both offline precomputation and online evaluation. All experiments were conducted on a Windows 10 laptop equipped with an Intel Ultra 9 185H CPU (2.5 GHz, 16 physical cores) and 32 GB RAM. The implementation uses Python 3.12 with NumPy 1.26, and no multi-threading or multi-processing optimizations are employed; all reported times reflect single-core execution.

#### 3.5.1. Offline Precomputation Scaling

The single-time-step effectiveness table is precomputed for varying numbers of devices (*I* = 7 to 12; four states each). Table 3 reports the state space size, construction time, and memory footprint. The results exhibit a linear trend with respect to $|\Omega_{X}|$ and remains practical: even for $4^{12}\approx 1.68\times 10^{7}$ states, the table is built in approximately 51 s and occupies only 64 MB.

#### 3.5.2. Online Evaluation Efficiency

The online evaluation cost depends on the number of non-zero probability entries in the posterior distribution rather than on the full state space size. Table 4 reports the average computation time for varying numbers of non-zero entries on the 7-device case study. Even with 10 million non-zero entries, the mapping completes in approximately 5 s; for typical DBN inference outputs, the overhead is negligible.

These results confirm that the framework-specific computation imposes negligible overhead, consistent with the modular architecture described in Section 2.5.2. The primary computational bottleneck remains the DBN inference itself, which is common to all probabilistic graphical model-based methods and can benefit from established approximate inference techniques.

## 4. Discussion

### 4.1. Comparison with Prior Work

To situate the proposed framework within the existing literature, Table 5 provides a systematic qualitative comparison against representative method categories across six distinguishing capabilities that are essential for pre-engagement defense effectiveness evaluation in ICS.

The proposed framework aims to simultaneously address all six capabilities within a single coherent model. This is achieved through two complementary mechanisms: the DBN structure, which encodes aleatoric uncertainty from dynamic state evolution and inter-device dependency propagation, and the explicit modeling of the defender’s partial observability, which captures epistemic uncertainty and conditions strategy evaluation on the defender’s actual information state. The value of this joint modeling approach, and the non-trivial interaction between the two uncertainty types it reveals, is discussed in Section 4.2.

### 4.2. The Value of Dual-Uncertainty Modeling

The ablation study in Section Ablation Study: Contribution of Uncertainty Sources quantifies the individual contribution of each modeling component. Beyond these isolated effects, the results reveal a deeper, non-additive coupling between epistemic and aleatoric uncertainty that only a joint modeling framework can capture. Two counterintuitive findings illustrate this interaction: (i) fixing the attack to the deterministic worst-case ($A^{1}$) yields higher prediction entropy than marginalizing over an unknown attack strategy ($A^{x}$) (Figure 13); (ii) replacing noisy observations with perfect state knowledge ($M_{\text{no-obs}}$) under an anomalous system state ($Z^{1}$) increases, rather than decreases, the predicted entropy (Figure 14).

These phenomena share a common mechanism. Epistemic uncertainty—whether arising from unknown attacker strategies or imperfect state observations—introduces a marginalization (or averaging) operation over heterogeneous risk pathways. Under $A^{x}$, the defender’s belief P(A∣X) averages over attack configurations ranging from benign to severe, blending distinct outcome distributions into a single, moderate-entropy predictive distribution. Under $A^{1}$, this averaging is eliminated: the defender conditions on a fixed, high-intensity attack, exposing the full aleatoric variability of state transitions and dependency propagation without the smoothing effect of cross-scenario averaging. Similarly, under noisy observations ($Z^{1}$), the posterior retains a low-probability “false-alarm” branch (healthy firewall) that carries substantially lower downstream uncertainty than the true-fault branch. Removing observation noise collapses the posterior exclusively onto the fault branch, stripping away the low-entropy mixture component and thereby elevating total entropy.

This interaction demonstrates that epistemic and aleatoric uncertainties do not simply superimpose; rather, they interact through the probabilistic structure of the model. Epistemic uncertainty can act as a dampening mechanism that partially conceals the true extent of aleatoric variability. Consequently, a framework that treats either source in isolation would systematically distort risk assessment: ignoring epistemic uncertainty (as in standard DBN methods that assume full observability) inflates apparent risk by exposing aleatoric variability that real defenders do not actually face; conversely, ignoring aleatoric uncertainty (as in deterministic game-theoretic models) underestimates the irreducible unpredictability of physical system evolution. The proposed DBN-based framework, by jointly encoding both uncertainty types within a single temporal probabilistic model, captures this coupling and provides a principled foundation for resilience-oriented, pre-engagement defense planning.

### 4.3. On the Choice of Entropy as a Companion Metric

Having introduced the dual-metric methodology, we now explain the rationale for coupling the expected effectiveness differential with information entropy, and discuss how this entropy-based companion metric complements and differs from alternative measures such as variance and worst-case metrics.

The expected value E(ΔF) and the variance Var(ΔF) are both moments of the distribution, computed as weighted sums over numerical outcome values. Variance therefore depends on the numerical encoding of system states: if device degradation levels were mapped to {0,1,2,3} versus {0,10,100,1000}, identical distributions would yield variance values differing by orders of magnitude. In ICS contexts where effectiveness values are elicited from domain experts and subject to revision, this encoding sensitivity is a practical liability. Entropy, computed as $-\sum p_{i}\log p_{i}$, depends solely on the probabilities of outcomes and is invariant to their numerical labels.

Beyond encoding independence, the ablation study reveals that entropy serves a diagnostic role that variance cannot replicate. Across all three ablation experiments, changes in entropy magnitude and direction quantitatively signal how each modeling component—attacker strategy, observation noise, and dependency propagation—constrains or expands the space of possible outcomes. In the dependency propagation case, entropy and variance move in opposite directions under anomalous observations (Section Ablation Study: Contribution of Uncertainty Sources), confirming that variance can misread structural changes that entropy correctly captures. This diagnostic sensitivity makes entropy a more reliable companion metric for frameworks that jointly model multiple uncertainty sources.

These diagnostic properties translate directly into strategy selection. Under anomalous observations, the entropy ranking diverges substantially from the expectation ranking (Section Strategy Evaluation with the Full Framework), demonstrating that strategies ranked highest by expectation may carry significantly higher predictive uncertainty. This confirms that entropy is not merely diagnostic but provides decision-relevant information that expectation-only evaluation would miss.

Tail-risk measures such as Value-at-Risk (VaR) and Conditional Value-at-Risk (CVaR) address yet another aspect of the distribution. Both the expected value and VaR/CVaR quantify the magnitude of effectiveness loss—the former in an average sense, the latter in extreme cases. Entropy answers a distinct question: “how confident are we in these predictions given the defender’s current information?” This dimension is orthogonal to loss magnitude and directly relevant to pre-engagement decision-making under incomplete observations, where defenders must choose strategies without knowing the true system state.

Crucially, the choice of entropy does not preclude the use of other metrics. Since the DBN inference procedure yields the complete posterior distribution of ΔF, any downstream risk measure can be directly computed from it. Incorporating variance or worst-case measures represents a natural extension fully compatible with the proposed framework. Exploring how the framework can be extended with conditional entropy or mutual information to explicitly quantify information asymmetry between attacker and defender represents a promising direction for future work [26].

### 4.4. Feasibility of Parameter Acquisition

A practical concern for deploying the proposed framework is the acquisition of the required parameters ($I_{1}$ to $I_{4}$). Obtaining accurate CPTs from real ICS environments is recognized as a difficult problem, due to data scarcity, security sensitivity, and the dynamic nature of threat landscapes. While the framework assumes known parameters for computation, in real-world systems defenders can construct or estimate these CPTs through existing technical pathways, as supported by current academic research and engineering practice:

- $I_{1}$: Utilizing long-term operational data combined with Bayesian network structure learning and parameter learning methods [21,27,28] to automatically infer the dependency topology between device states and estimate their conditional probability distributions from historical data. This approach has mature theoretical support in the field of probabilistic graphical models.
- $I_{2}$: Based on alerts and audit logs from monitoring systems (e.g., SIEM), this precisely quantifies the mapping relationship between the true state and the observed state (e.g., false positive rate, false negative rate).
- $I_{3}$: This parameter represents the defender’s belief about “which strategies the attacker tends to adopt under specific system states.” Although the defender cannot directly know the attacker’s strategy distribution, this is essentially a threat behavior modeling problem and can be constructed in two ways: (1) Prior modeling based on authoritative knowledge bases and attack frameworks: defenders can leverage public, standardized cybersecurity knowledge bases, such as the MITRE ATT&CK ICS framework [29], to map the attack techniques in the framework to the attack strategy set in this model. Then, based on the preconditions for attack techniques described in the framework (e.g., required privileges, dependencies on system states) and combined with analyses of system vulnerabilities from guides like NIST SP 800-82 [20], they can qualitatively estimate under which system states a particular attack technique is more likely to be triggered. (2) Data-driven correction based on historical security events and threat intelligence: defenders can analyze internal historical security logs and external threat intelligence reports, using log mining and correlation analysis techniques [30,31] to count the frequency of different attack tactics and techniques occurring under specific system states, thereby using data to calibrate and enrich the prior model based on knowledge bases.
- $I_{4}$: This parameter quantifies the likelihood of system state transitions under specific attack–defense strategy combinations, fusing attack effectiveness and defense effectiveness. Defenders can leverage public vulnerability databases (e.g., CVE/NVD), using CVSS exploitability scores as proxy variables for estimating attack success probability; alternatively, by repeatedly executing specific attack–defense actions in highly realistic ICS testbeds [32,33], the frequency of state transitions can be statistically obtained to estimate their probability.

### 4.5. Practical Deployment Workflow

To facilitate the adoption of the proposed framework in real ICS environments, we outline a practical deployment workflow consisting of five steps.

1. **DBN construction and maintenance.** The defender constructs the complete DBN—including true state variables X, observation variables Z, attacker strategy variables A, and defender strategy variables D—from system architecture documentation, control logic specifications, network topology diagrams, and the deployed monitoring infrastructure. The DBN structure should be reviewed and refined after major system upgrades or topology changes.
2. **CPT specification.** The four types of CPTs are populated through the pathways discussed in Section 4.4: prior distributions from operational data or device reliability statistics, observation likelihoods from SIEM false positive and false negative rates, attacker strategy models from the MITRE ATT&CK for ICS framework and threat intelligence, and state transition probabilities from vulnerability databases or testbed experiments.
3. **Effectiveness function and offline precomputation.** The defender defines the state variables contributing to system effectiveness, assigns the device weights and single-device effectiveness functions according to organizational priorities, and precomputes the single-time-step effectiveness table for all single-time-step state configurations. This table is reused across all subsequent online evaluations (Section 2.5).
4. **Online evaluation.** During pre-engagement planning, the defender supplies the current observation vector and a candidate defense strategy. The DBN inference engine computes the posterior joint distribution, and the effectiveness evaluation module outputs E(ΔF) and H(ΔF) as described in Section 2.5.
5. **Periodic model maintenance.** The DBN structure and CPTs should be reviewed and updated after major system reconfigurations, significant threat landscape changes, or monitoring tool upgrades. The observation model merits particular attention, as changes in sensor deployment or IDS rule sets can shift false positive and false negative rates. The perturbation analysis (Section 3.4.2) demonstrates that the framework tolerates up to 15% parameter drift, so continuous fine-tuning is unnecessary.

### 4.6. Defender-Attacker Duality

The DBN formalism introduced in this work possesses a natural structural symmetry between the defender’s and the attacker’s perspectives. In the graphical structure shown in Figure 2, the defender’s observation Z(t) and the attacker’s observation Y(t) occupy symmetric positions as evidence nodes conditioned on the true system state X(t), while the defense strategy D(t) and the attack strategy A(t) are symmetrically positioned as decision nodes that influence the state transition to X(t+1). This duality is not an incidental feature but a direct consequence of modeling the attack–defense interaction as a closed-loop dynamic game within a single temporal probabilistic framework.

A practical implication of this symmetry is that the same computational machinery developed here for pre-engagement defense evaluation can, with a straightforward exchange of input roles, support offensive campaign planning in red-team contexts. An attacker equipped with the same DBN model could evaluate candidate attack strategies under different assumptions about the defender’s observation capabilities and likely defensive responses. This dual-use capability is enabled by the modular separation of the DBN inference engine from the effectiveness function and the observation models, allowing either party’s perspective to be instantiated without modifying the underlying probabilistic graphical structure.

### 4.7. Limitations and Future Work

Despite the promising results, several limitations of the current study should be acknowledged, each pointing to a corresponding direction for future investigation.

The experimental evaluation is conducted on a single substation automation system using simulated CPTs based on domain knowledge, with a finite set of observed states and defense strategy combinations, and has not been validated against data from a real ICS deployment. While the CPT perturbation analysis (Section 3.4.2) provides evidence that the framework’s qualitative findings are robust to parameter uncertainty, confirming the transferability of these findings to operational environments remains an important direction for future work. Extending the assessment to additional ICS domains (e.g., water treatment, oil refineries), a broader range of observed states and defense strategy combinations, and to real-world operational data would help bridge the gap between simulation-based and real-world evaluation results.

The proposed framework requires four types of CPTs ($I_{1}$ through $I_{4}$) as input. In the absence of sufficient operational data for learning, these CPTs are, in the current case study, specified by domain experts. Section 4.4 discusses several technical pathways for parameter acquisition in operational settings; however, each of these methods faces practical barriers and their effectiveness has not yet been demonstrated in conjunction with the proposed framework. Developing and validating data-driven methods for CPT calibration from real ICS environments thus constitutes an important direction for future work.

The computational scalability experiments (Section 3.5) verify that the framework-specific computation is not a bottleneck. However, the full DBN inference pipeline has not been stress-tested on networks with hundreds of nodes, where exact inference becomes prohibitive. While the modular architecture allows mature approximate inference techniques to be directly substituted without modifying the evaluation module, this extension has not yet been empirically validated and represents an important direction for future work.

The framework assumes that the defender models the attacker as a cognitively rational Bayesian decision-maker, grounded in the belief that the attacker’s strategy is a function of the current true system state. Specifying a more generalized attacker model that accounts for non-Bayesian or boundedly rational decision-making processes represents a promising extension. This requires the defender to manage an additional layer of epistemic uncertainty—uncertainty about the attacker’s cognitive model itself—which could be addressed through a hierarchical Bayesian framework that accommodates multiple candidate attacker models. In this framework, the defender would maintain a belief over possible adversary cognitive archetypes and marginalize over them during strategy evaluation, thereby providing robust defense recommendations that hedge against misspecification of the attacker’s decision logic.

## 5. Conclusions

This paper addressed the challenge of quantitatively evaluating defense strategy effectiveness in ICS under dual uncertainty. We proposed a DBN based framework that jointly models epistemic and aleatoric uncertainty within a single temporal probabilistic model, and a dual-metric evaluation methodology coupling expected effectiveness with information entropy.

A systematic qualitative comparison against six representative method categories demonstrated that no existing framework simultaneously addresses all six distinguishing capabilities required for pre-engagement ICS defense evaluation. A case study on a substation automation system, complemented by systematic ablation experiments, demonstrated the framework’s ability to distinguish defense strategies and to reveal structural dependencies that contradict conventional device-centric intuition. Robustness analysis confirmed stable dual-metric evaluation under up to 15% noise in CPTs. The ablation study demonstrated that observability, dependency propagation, and attacker strategy uncertainty each contribute distinctively to total prediction entropy. Crucially, reducing epistemic uncertainty can unmask latent aleatoric variability. These results provide theoretical and methodological foundations for resilience-oriented cyber defense planning in ICS.

## Figures and Tables

**Table 1:** Summary of key notation used in the DBN-based evaluation framework.

Symbol	Description
System State & Observations	
X/X(t)	True system state vector (general/at time *t*)
Z/Z(t)	Defender’s observation vector
Y/Y(t)	Attacker’s observation vector
$X_{i}$/$X_{i}\left(t\right)$	State of device *i* (scalar component of X)
x	A specific realization (value) of X
$\Omega_{X}$	Sample space of the true system state
Strategy & Actions	
A(t)	Attacker’s strategy vector at time *t*
D(t)	Defender’s strategy vector at time *t*
$A_{i}\left(t\right),D_{j}\left(t\right)$	Binary indicators for specific attack/defense strategies
Probabilistic Dependencies	
P(X∣Z)	Defender’s posterior belief about the true state given observations
P(X∣Y)	Attacker’s posterior belief about the true state given observations
P(A∣X)	Defender’s belief about the attacker’s strategy given the true state
Effectiveness Metrics	
φ(X)	System effectiveness function
ΔF	Effectiveness differential: φ(X(t+1))−φ(X(t))
E(ΔF)	Expected effectiveness differential
H(ΔF)	Information entropy of the effectiveness differential
Bayesian Network Notation	
par(v)	Set of parent nodes of random variable *v*
par(V)	Union of parent node sets for all v∈V

**Table 2:** Expected effectiveness and strategy gains under $Z^{2}$ for $M_{\mathrm{full}}$ and $M_{\text{no-dep}}$.

Strategy	$M_{\mathrm{full}}$		$M_{\text{no-dep}}$	
	E(ΔF)	Gain	E(ΔF)	Gain
$D^{0}$	−0.2181	—	−0.2744	—
$D^{1}$	−0.1492	+0.0689	−0.2461	+0.0283
$D^{3}$	−0.1521	+0.0660	−0.2258	+0.0486
$D^{1,3}$	−0.0749	+0.1432	−0.1975	+0.0769
$D^{1}+D^{3}$ (sum)	—	+0.1349	—	+0.0769

**Table 3:** Offline precomputation performance.

*I*	$|\Omega_{X}|$	Time (s)	Memory (KB)
7	16,384	0.04	64
8	65,536	0.15	256
9	262,144	0.65	1024
10	1,048,576	4.08	4096
11	4,194,304	16.18	16,384
12	16,777,216	51.49	65,536

**Table 4:** Online evaluation time vs. number of non-zero probability entries (*I* = 7).

Non-Zero Entries	Time (s)
100,000	1.26
1,000,000	1.35
5,000,000	3.21
10,000,000	5.08

**Table 5:** Qualitative comparison of the proposed framework against existing method categories.

Method Category	PO	DE	DP	EU	AU	Entropy
Static BN [6,7]	×	×	✓	×	✓	×
Dynamic Assessment [8,9,18]	×	✓	✓	×	✓	×
Game-theoretic/Optimization [10,11,12]	×	✓	× (Ltd.)	×	✓	× (Part.)
Attack Prediction and Detection [13,14]	✓ (Part.)	✓	✓	✓ (Part.)	✓	×
POMDP-based Resilience [19]	✓	✓	×	✓	✓	×
Uncertainty Quantification [15,16,17]	✓ (Part.)	× (Ltd.)	×	✓ (Part.)	✓ (Part.)	× (Part.)
**This Work (DBN + Entropy)**	✓	✓	✓	✓	✓	✓

**PO:** partial observability; **DE:** dynamic evolution; **DP:** dependency propagation; **EU:** epistemic uncertainty; **AU:** aleatoric uncertainty; **Entropy:** information entropy adopted as a companion metric. “Ltd.” = limited; “Part.” = partial. “Ltd.” for dynamic evolution in the uncertainty quantification row indicates that Ref. [17] models continuous-time dynamics via differential games, whereas Refs. [15,16] employ static models. “Ltd.” for Dependency Propagation in the Game-theoretic row reflects that these methods model attack paths through network topology but do not explicitly capture ICS-specific functional dependencies among devices. “Part.” in the Game-theoretic row indicates that Ref. [10] uses entropy only for single-dimensional strategy uncertainty. “Part.” for Attack Prediction and Detection reflects that only Ref. [14] addresses PO and EU (via its HMM-based MMSE detector). “Part.” for Uncertainty Quantification indicates that Ref. [15] uses entropy for weight calculation, while Refs. [16,17] do not employ entropy as a metric.

## Data Availability

The simulation data used in this study are available at https://github.com/engutou/ICS-Effectiveness (accessed on 9 April 2026).

## Abbreviations

ICS	Industrial Control Systems
DBN	Dynamic Bayesian Network
CPT	Conditional Probability Table
SCADA	Supervisory Control and Data Acquisition
IDS	Intrusion Detection System
